# Harmful Leader Behaviors: Toward an Increased Understanding of How Different Forms of Unethical Leader Behavior Can Harm Subordinates

**DOI:** 10.1007/s10551-021-04864-7

**Published:** 2021-06-28

**Authors:** Juliana Guedes Almeida, Deanne N. Den Hartog, Annebel H. B. De Hoogh, Vithor Rosa Franco, Juliana Barreiros Porto

**Affiliations:** 1 Amsterdam Business School, University of Amsterdam, Leadership & Management, P.O. Box 15953, 1001 NL Amsterdam, Netherlands; 2 Department of Psychology, São Francisco University, Rua Waldemar César da Silveira, 105, Jardim Cura D’ars (SWIFT), Campinas, São Paulo, Brazil; 3 Department of Social and Organizational Psychology, University of Brasilia, Universidade de Brasília, Campus Darcy Ribeiro, ICC Sul, sala AT-013, Brasília, Brazil

**Keywords:** Harmful leader behavior, Unethical leadership, Destructive leadership, Scale development

## Abstract

Research on unethical leadership has predominantly focused on interpersonal and high-intensity forms of harmful leader behavior such as abusive supervision. Other forms of harmful leader behavior such as excessively pressuring subordinates or acting in self-centered ways have received less attention, despite being harmful and potentially occurring more frequently. We propose a model of four types of harmful leader behavior (HLB) varying in intensity (high vs low) and orientation (people/relationships or tasks/goals): Intimidation, Lack of Care, Self-Centeredness, and Excessive Pressure for Results. We map out how these relate to other constructs in the unethical leader behavior field in order to integrate the existing work on how leaders can cause harm to followers. Next, in five studies (*N* = 35, *N* = 218, *N* = 352, *N* = 160, *N* = 1921 in 196 teams), we develop and test a new survey instrument measuring the four proposed types of perceived HLB. We provide initial validity evidence for this new measure, establish its psychometric properties, and examine its nomological network by linking the four types of HLB to related leadership constructs and soft and hard outcome correlates at the individual and team level. We find that HLB is negatively related to constructive forms of leadership (e.g., ethical and transformational) and positively to unethical ones (e.g., abusive supervision). HLB is also related in the expected direction to job satisfaction, engagement, psychological safety, knowledge sharing, knowledge hiding, deviance, and objectively recorded team-level stress-related absenteeism.

Recent work on leadership shows a growing interest in unethical leader behaviors that are harmful to subordinates (Einarsen et al., 2007). Research to date has mostly focused on nonphysical forms of abuse and interpersonal aggression displayed by the leader, such as angry outbursts, public ridiculing, and openly attacking subordinates (Tepper et al., 2017). Evidence has been accumulating that such behaviors are detrimental to followers and organizations (Schyns & Schilling, 2013). However, overtly misbehaving, ranting managers are not the only concern. Most scholars agree that unethical leadership is a complex and multidimensional phenomenon as leaders can also be harmful to subordinates in other, less intense ways (cf. Thoroughgood et al., 2012). For example, Mitchell and Ambrose (2007) propose there are passive and aggressive forms of abusive supervision and we know that leaders may also be harmful by bullying or showing a lack of care for followers (Einarsen et al., 2007).

Abusive supervision forms an influential, albeit low base rate phenomenon (e.g., Zellars et al., 2002). Yet, Hogan and Kaiser (2005) estimate that two thirds of managers are bad or ineffective leaders. Others suggest leaders regularly exploit followers or neglect their leadership responsibilities (e.g., Einarsen et al., 2007; Schmid et al., 2019). Given that such less intensive behaviors may be less visible, they may be more common and more tolerated than abusive supervision. Also, while such less intense behaviors have received far less attention in unethical leadership research to date than high-intensity ones (cf. Einarsen et al., 2007), they do merit being investigated as these behaviors can cause harm to subordinates.

Here, we aim to integrate previous work on different ways in which leaders can be harmful to their followers, and following classical leadership theory (e.g., Judge et al., 2004), we suggest that leaders are not only harmful in the interpersonal domain, such as through abusive supervision, but can also be harmful via task- or goal-oriented behaviors, such as overloading subordinates or pursuing selfish goals. We develop a model of harmful leader behavior (HLB) that distinguishes between four types of harmful behavior differing in intensity (high-low) and orientation (people/relationship task/goal). These four types are labeled Intimidation, Lack of Care, Self-Centeredness, and Excessive Pressure for Results and the model is depicted in Fig. 1. In this figure, we also show the “conceptual neighborhood” (cf. Meuser et al., 2016) of the four HLBs and below we discuss how HLBs relate to and differ from other unethical forms of leader behavior that have been addressed in the field (see also Table 1).

**Fig. 1:**
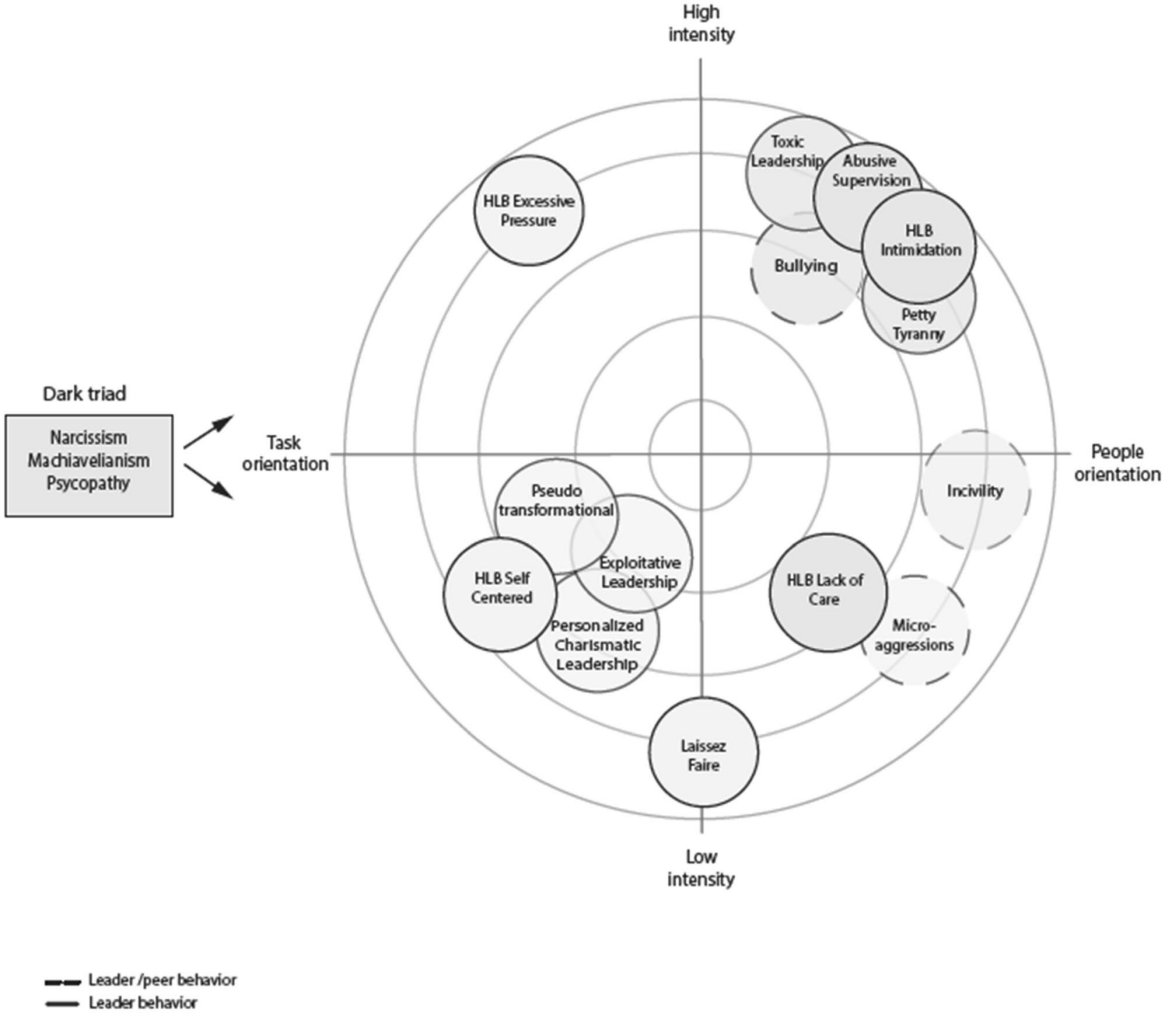
Theoretical model and conceptual neighborhood of harmful leader behavior

In addition to developing the conceptual framework, we present a measure for the four types of HLB. Existing scales typically do not assess harmful leadership as a multidimensional phenomenon. These measures either focus predominantly on intense and people-oriented unethical leader behavior, or measure both high- and low-intensity behaviors mixed in a single scale, without distinguishing specific dimensions to assess whether they have similar or different correlates. Also, some existing measures focus on more general harmful workplace behavior or criminal behavior, which we do not include. Here, we add task/goal-oriented forms to the interpersonal ones and develop four scales to measure these. In sum, we propose four types of harmful leader behavior that can be perceived by followers, we integrate these into the leadership and ethics literature, and we develop and validate a new instrument to measure these HLBs in five studies.

## Harmful Leader Behavior: Intensity and Orientation

The literature on unethical leadership suggests that employee perception is key to the effects of destructive and unethical forms of leadership (see e.g., Schyns & Schilling, 2013) and in line with this we focus on assessing followers’ HLB perceptions. We define perceived harmful leader behavior as followers’ perception of their leaders’ misuse of power and display of behaviors inflicting recurring or enduring harm to their followers. We use the term harmful to clarify that the model does not attempt to include all possible forms of leader behavior that might fall under the broader labels of unethical or destructive, and we focus specifically on behaviors that are harmful to subordinates (rather than the organization or other stakeholders such as clients or society).

Previous work on such harmful and unethical leader behavior has often been rooted in the work on workplace aggression (Neuman & Baron, 1998). Yet, most of the work to date has focused on high-intensity leader behavior (cf. Schyns & Schilling, 2013; Thoroughgood et al., 2012), while workplace abuse most of the time is subtle and indirect (e.g., Keashley, 1998). Low-intensity forms of harmful leadership have received less attention, even though several authors recognize that leaders may use harmful means even when the intent to harm is not evident (Tepper et al., 2017), and the wider literature suggests that both high- and low-intensity behaviors can cause harm to others (e.g., Neuman & Baron, 1998). Low-intensity behaviors are more easily denied and, as they are more veiled, they are harder to give meaning to (Keashley, 1998). Thus, instigators can more easily claim that they didn’t know this behavior could harm or that their intentions were misinterpreted. Less visible low-intensity behaviors may require more occurrences to be recognized and more awareness from followers than high-intensity ones.

Besides the difference in intensity, classic leadership theory suggests that leaders’ (positive) behaviors generally are people-oriented (also labeled relationship oriented, consideration) or goal-oriented—(also labeled task-oriented or initiating structure) (e.g., Judge et al., 2004; Lambert et al., 2012). People-oriented leaders show they care about interpersonal factors and subordinates’ needs. Goal- or task-oriented leaders manage goal achievement and show subordinates how to best accomplish tasks (Bass & Bass, 2008). These two basic orientations have somewhat different correlates and outcomes (Bass & Bass, 2008). Leaders’ task-oriented behavior is more highly correlated with leader and team performance (Judge et al., 2004), while people-oriented behavior is more related to satisfaction at work and group maintenance (e.g., Fisher & Edwards, 1988). People-oriented behavior is also more strongly related to employee outcomes such as motivation, leader satisfaction, OCB and trust in the leader than task-oriented behavior (Judge et al., 2004; Lambert et al., 2012). We propose that the basic distinction also applies to the dark side of leadership, namely to harmful leader behavior.

On the dark side, research has extensively looked at person-oriented (and high intensity) harmful behavior through the lens of abusive supervision (Tepper et al., 2017). However, to date, harmful task/goal-oriented forms of behavior have mostly been neglected. Task focused HLB may include leaders claiming others’ successes as their own or placing excessive demands on employees such as pressuring them to work more than is reasonable. Some early research indeed suggested that leaders can display harmful task related behaviors (e.g., creating work overload, assigning tasks as punishment, unfair performance assessments on purpose). More recent work sometimes considers overloading subordinates as predictive of abusive leadership (Flores et al., 2016) or as undermining subordinates (Greenbaum et al., 2012), but also looks at positive outcomes (Babalola et al., 2020). To date task- or goal-oriented behaviors have not been conceptualized or studied as forms of HLB per se as we do here. In our model, we distinguish both high versus low intensity and people versus task/goal-oriented HLB (see Fig. 1) and we map out related constructs to depict the “conceptual neighborhood” (cf. Meuser et al., 2016) of the four HLBs. Below we describe all four HLBs and show how they are similar to and different from related constructs (see also Table 1).

## A Constitutive Definition of the Four Proposed HLBs

Four basic types of harmful behavior (see Fig. 1) result from the combination high versus low intensity and people versus task-oriented leader behavior. We label these Intimidation, Lack of Care, Self-Centeredness and Excessive Pressure for Results.

### Intimidation

Intimidation is a HLB that focuses on high-intensity public interpersonal maltreatment of subordinates (people-oriented harmful leader behavior). We define Intimidation as the public display of undermining and punishing behaviors that humiliate targets and place them in a submissive, powerless position. Research shows that such leader behavior affects subordinates negatively. For example, abusive supervision (the closest variable to Intimidation in the field, see Table 1) is negatively related to employee performance and well-being (Tepper, 2007; Walter et al., 2015).

### Lack of Care

Lack of Care is an interpersonally focused form of low-intensity HLB. It involves a display of sustained lack of concern for subordinates’ needs and well-being (Skogstad et al., 2007). This runs counter to the core of leader people orientation or consideration (Bass & Bass, 2008). A lack of care often implies leaders being aloof, unsupportive, and not taking leadership responsibility. Such indirect and uncaring behaviors have a negative impact on subordinates’ affective commitment, work effort, and citizenship (e.g., Buch et al., 2015). They can increase role conflict, role ambiguity, and conflict among coworkers and thus Skogstad et al. (2007) suggest that not supporting and not caring about subordinates welfare are harmful behaviors (see also Thoroughgood et al., 2012). We define the low-intensity interpersonal form of harmful leader behavior as subordinates’ perceptions that leaders lack care about subordinates’ needs or well-being and do not value team members.

### Self-Centeredness

Low-intensity task-oriented HLB occurs when leaders prioritize their personal interests over collective goals. The relationship between leaders and subordinates should reflect a social contract in which leaders agree to pursue actions that are in the collective best interest and to steer the team in this direction (Maner & Mead, 2010). However, Maner and Mead found that leaders often prioritize personal over team goals, protecting their privileged position. Indeed, a reference to leaders’ self-interest is recurrent in leadership research (Schmid et al., 2019). Prioritizing personal interests rather than or even at the expense of those of the team implies the leader goes against the social contract, which is harmful (Cramwinckel et al., 2013).

Leaders control the means at work and can opt to use their power purely for the sake of self-interest. They can manipulate people to achieve personal goals and behave in a self-centered manner. Taking credit for work done by others in the team, manipulating people, taking privileges to maximize self-interests and favoring team members who can contribute more to personal projects are examples of such low-intensity, self-centered goal or task-oriented behaviors that can end up harming subordinates. Self-Centeredness is defined as the display of leader behavior that serves the self-interest of the leader at the expense of the team (members). Such behavior affects subordinates negatively (decreasing job satisfaction and commitment, and increasing burnout and deviance, see e.g., Schmid et al., 2019).

### Excessive Pressure for Results

Excessive pressure combines high-intensity and task- or goal orientation and has not yet been studied as an unethical form of leadership. This form of behavior implies that the means the leader uses to steer subordinates toward accomplishing tasks or team goals are harmful to subordinates. For example, when leaders are applying excessive pressure to perform, frequently overloading employees, exclusively focusing on performance at the expense of other aims and subordinate welfare, or placing unreasonable demands on team members. Leaders might be tempted to use these harmful task-oriented means to reach results “as leaders often must go to extremes to meet tough challenges” (Kaplan & Kaiser, 2003, p. 19) and this urgent need can overshadow the morality of the process to achieve results and the negative impact this behavior has on the team and its members (Ma et al., 2004).

The literature highlights that goal setting and especially a strong pressure for performance can have a dark side as this can foster unethical behaviors such as cheating, deception and manipulation (e.g., Niven & Healy, 2016). For example, the will to be promoted or receive a higher bonus might influence leaders to neglect competing priorities or ethical guidelines and focus on the bottom line as the only relevant outcome. Leaders may then “care more about profits than employee well-being” (Greenbaum et al., 2012, p. 359) and perceive that the ends justify the means. Thus, we focus on excessive pressure for results, as a high-intensity task- or goal-focused harmful leader behavior and define it as the display of behaviors strategically directed to increase team performance and meet task goals at any cost, disregarding subordinates’ well-being and quality of work life.

Below, we develop a multidimensional measure that distinguishes between high and low intensity and people and task/goal-oriented HLB, but first we discuss how the proposed constructs relate to previous conceptual work and existing measures.

## HLB and Prior Unethical Leadership Research

Earlier work has introduced many different constructs and measures in the area of unethical leadership (e.g., Schyns & Schilling, 2013, see Table 1 and Fig. 1 for the most prominent examples of these). To address how the four behaviors in our model and measure relate to previous work in the field, we identified several constructs that partially overlap with three of the HLB subscales. These previous constructs and scales have some similarities. First, all are operationalized as resulting in psychological harm at work (e.g., workplace violence and aggression, Neuman & Baron, 1998; incivility, Cortina et al., 2001; destructive leader behavior, Thoroughgood et al., 2012). Second, all use subordinates’ perceptions of leader behavior to assess the phenomena. Third, these phenomena are all described as recurrent and unwelcome ways of acting by an individual. Finally, with the exception of the concept of incivility (Cortina et al., 2001) which assigns the instigator a lack of clear conscious intention and laissez faire which entails not taking action (Bass & Avolio, 2000), the behaviors generally are seen as being displayed voluntarily. HLBs share these characteristics with this previous work, but there are also differences.

**Table 1:** Unethical leadership constructs and measures in comparison to the four HLBs

Constructs	Definition	Multi-dimensional	Type of harm	Power differences	High or low intensity	People or goal orientations	Results in positive outcomes	Criminal activity included	Similarities and differences with HLB phenomena
Pseudo-transformational (Bass & Avolio, 1997) Measured with a specific profile on the MLQ; see Christie et al., 2011)	Pseudo-transformational leaders focus on their self-interest and status in an organization and will exploit transformational behaviors to make followers comply (Bass & Avolio, 2000)	Profile on MLQ	Psychological	Yes	Low intensity	Task oriented	Potentially	No	Neighbor: HLB self-centeredness. Difference: pseudo-transformational leaders focus on personal gain through the misuse of transformational influence, whereas HLB self-centeredness taps specific self-serving task related behaviors
Personalized charismatic leadership (Popper, 2002) 6 items	Personalized charismatic leaders have a desire to accumulate personal power and strive to elicit follower commitment and loyalty and are manipulative and exploitative of others, disregarding their feelings (House & Howell, 1992)	No	Psychological	Yes	Low intensity	Task oriented	Potentially	No	Neighbor: HLB self-centeredness. Difference: like HLB self-centeredness personalized charismatic leaders focus on self-serving goals. However, they do so through inducing follower’s identification based on the attractiveness and referent power of the leader, rather than specific self-serving task related behaviors
Abusive supervision (Tepper, 2000) 15 items	Sustained display of hostile verbal and nonverbal behaviors, excluding physical contact	No	Psychological	Yes	High intensity	People oriented	No	No	Neighbor: HLB intimidation. Difference: abusive supervision is usually described as a high-intensity behavior (Tepper et al., 2017) though previous literature suggested the full measure includes both high and low intensity behaviors in the same scale. HLB Intimidation exclusively taps high-intensity behavior
Bullying in the workplace—negative acts questionnaire-revised; (Einarsen et al., 2009) 22 items	The persistent exposure to interpersonal aggression and mistreatment from colleagues, superiors or subordinates	Yes	Psychological and physical	Not necessarily	High intensity	People and task oriented mixed	No	No	Neighbor: HLB intimidation. Difference: workplace bullying is a measure of general workplace misbehavior toward an employee that can stem from different sources, not just the leader. It also includes physical aggression. Though conceptualized as high-intensity behavior, the measure includes both low- and high-intensity behaviors. HLB Intimidation is specifically focused on high-intensity behavior and does not include physical aggression
Incivility (Cortina et al., 2001) 7 items	Low-intensity deviant behavior with ambiguous intent to harm the target, in violation of workplace norms of mutual respect. Uncivil behaviors are characteristically rude and discourteous, displaying a lack of regard for others	No	Psychological	Not necessarily	Low intensity	People oriented	No	No	Neighbor: HLB lack of care. Difference: uncivil behaviors can be displayed by both leaders and coworkers. Conceptualized as low intensity behavior, the concept is partially operationalized with high-intensity behaviors. Items somewhat relate to HLB Intimidation, with little to intermediate overlap with both the people-oriented HLBs
Laissez faire—MLQ (Bass & Avolio, 2000) 10 items	An inactive leadership style Laissez faire leaders do not take leadership responsibility, are passive and seek to avoid their leadership duties and their subordinates	No	Psychological	Yes	Low intensity (avoidance)	People and task oriented mixed	Potentially	No	Neighbor: HLB lack of care. Difference: the MLQ laissez faire scale measures low-intensity behavior operationalized as avoidance of leadership responsibility, passivity, and non-behavior. HLB Lack of care addresses low-intensity person-oriented behaviors
Micro aggressions (e.g., Sue et al., 2007; Molero et al., 2013) Multiple scales	Micro aggressions describe subtle forms of discrimination of minorities motivated by for example racist or (hetero)sexist attitudes	Depends on measure	Psychological	Not necessarily	Low intensity	People oriented	No	No	Neighbor: HLB lack of care. Difference: microaggression refers to subtle forms of discrimination that can be perpetrated by leaders, colleagues and subordinates. Focus on aggressions of marginalized groups. HLB lack of care more specifically refers to leader behavior, regardless of subordinates’ minority status
Workplace violence and aggression (Neuman & Baron, 1998) 32 items	Workplace aggression as efforts by individuals to harm others with whom they work, or have worked, or the organization in which they are presently, or were previously, employed	Yes	Psychological and physical	Not necessarily	Low- and high-intensity mixed	People oriented	No	Yes	Neighbors: multiple HLB scales. Difference: this multidimensional measure includes both high and low-intensity behaviors. Crimes, some more general behavior (nonspecific to leaders) and avoidance are also part of the scale. HLBs each focus on a specific quadrant, and do not include crimes
Destructive leader behavior (DLB; (Thoroughgood et al., 2012) 26 items	DLB focuses on the leader’s voluntary engagement in a broad array of harmdoing and destructive behavior. DLB is voluntary in that the leader either lacks the motivation to act in a constructive fashion or becomes motivated to act in harmful ways (Robinson & Bennett, 1995)	Yes	Psychological and physical	Yes	Low- and high-intensity mixed	People and task oriented mixed	No	Yes	Neighbor: multiple HLB scales. Difference: multidimensional measure that includes both high- and low-intensity behaviors. Crimes/sexual harassment and avoidance are also included in the scale. Some items describe more general behaviors (nonspecific to leaders). The behaviors tapped are more people-oriented with a few items oriented to the task. HLBs each focus on a specific quadrant, and do not include crimes

Several previous measures of destructive or unethical forms of leadership include criminal leader behaviors, such as harmful physical contact, violence, sexual harassment or fraud. Such criminal behaviors are much less recurrent and need legal punishment, which is not our focus here. Also, while we separate high vs low intensity and person vs task orientation, most previous measures include items that mix orientations and intensity levels. Finally, several measures also address more general observed behavior that could be displayed by different stakeholders, not necessarily only the leader, whereas the measure of HLBs we present below focuses specifically on leader behavior. Table 1 displays an overview of most prominent previous scales and relevant differentiating characteristics.

Figure 1 maps the conceptual space around HLBs. As noted, we see that research on unethical leadership has predominantly focused on interpersonal and high-intensity forms of harmful leader behavior such as abusive supervision (Tepper et al., 2017), toxic leadership (Schmidt, 2008), bullying (Einarsen et al., 2009) and petty tyranny (Ashforth, 1994). Several low-intensity task-oriented forms of unethical leader behavior such as pseudo-transformational (Bass & Avolio, 1997), personalized charismatic leadership (Popper, 2002) as well as exploitative leadership (Schmid et al., 2019) have also been identified, but have received less research attention as have interpersonally focused forms of low-intensity behavior (cf. incivility, Cortina et al., microaggressions, Sue et al., 2007). High-intensity task- or goal orientation forms of unethical leader behavior have also hardly been addressed. Below, without being exhaustive, we discuss several of these previous constructs and scales in more detail, comparing them to the HLBs (see also Table 1).

In addition to leader behavior, the unethical leadership literature also looks at the role of negative or dark triad traits of narcissism, psychopathy and Machiavellianism (e.g., Jonason & Webster, 2010). Rather than forming behaviors in themselves, these traits are likely to be important predictors of different harmful (leader) behaviors. For example, conceptually both narcissism and Machiavellianism should predict self-centered leader behavior and research has also linked both of these traits to abusive supervision (e.g., Kiazad et al., 2010; Nevicka et al., 2018).

### HLBs and Abusive Supervision

Most research on unethical leader behavior to date has focused on abusive supervision (Tepper et al., 2017). With content that overlaps strongly with abusive supervision (Tepper, 2000), we conceptualize the HLB of Intimidation as abusive leader behavior which includes publicly undermining, blaming, or humiliating subordinates and displaying outbursts of aggression (Tepper et al., 2017). Both HLB Intimidation and abusive supervision represent high-intensity and people-oriented leader behavior. However, abusive supervision has an active and a more passive side (Mitchell & Ambrose, 2007) and both are represented in the full 15-item scale. The HLB Intimidation scale exclusively focuses on high-intensity behavior. Also, some abusive supervision items describe more general behavior that could be displayed by anyone in the organization while HLB Intimidation focuses only on leader behavior and provides a more focused measure.

### HLBs and Workplace Bullying

Bullying in the workplace can be perpetrated by leaders, but also by colleagues or subordinates. Though conceptualized as high-intensity people-oriented behavior, items include both high and low-intensity behaviors. Also, people and task-oriented items are mixed in the scale, which also includes more general workplace behavior (Einarsen et al., 2009—“being ignored or excluded”).

### HLBs and Pseudo-Transformational Leadership

Pseudo-transformational leaders misuse their influence on followers exploiting them (Bass & Bass, 2008). Pseudo‐transformational leadership describes self‐serving, yet highly inspirational leader behavior, combined with an unwillingness to encourage independent thought in followers, and little care for them (Barling et al., 2008; Christie et al., 2011). Pseudo-transformational leaders are proposed to be first and foremost self-serving, focused on themselves and their needs. As a construct this type of leadership forms a conceptual neighbor of Self-centered HLB. Yet it is a broader construct, also including inspirational behavior. HLB Self-centered is thus a more focused measure for specific task related self-serving leader behaviors.

### HLBs and Personalized Charismatic Leadership

As personalized charismatic leaders have a desire to accumulate power and manipulate followers in order to achieve personal goals (House & Howell, 1992), they also behave in self-serving ways, which makes them a conceptual neighbor of self-centered HLB. However, like pseudo-transformational leadership, personalized charismatic leadership is a broader construct which also addresses charismatic influence.

### HLBs and Incivility

Incivility is a low-intensity workplace behavior with no clear intent to harm. While half the items are focused on low-intensity behavior, the construct is also partially operationalized with behaviors that are similar to the ones encompassed by measures of higher intensity such as abusive supervision and Intimidation (Cortina et al., 2001—e.g., “Put you down or was condescending to you”? and “Made demeaning or derogatory remarks about you”?). Therefore, incivility presents content that is related to both HLB Intimidation and Lack of Care. In addition, incivility focuses more generally on harmful workplace behavior that can be displayed by leaders but also by others.

### HLBs and Laissez Faire

Though leadership implies influence, laissez faire leaders are described as leaders that display an overall avoidance to lead or influence others (Bass & Bass, 2008). It is most closely related to HLB Lack of Care as it assesses a lack of adequate (relational) leadership. Yet, the HLB items assess behaviors, while laissez faire items describe non-behaviors (an example of a Laissez faire item is: “avoids making decisions”) as such leaders are inactive. Also, laissez faire is a somewhat broader construct that measures being passive and an avoidance in both people and goal-oriented ways (e.g., “has avoided telling me how to perform my job”), while HLB Lack of Care specifically focuses on low-intensity people-oriented behavior, such as demonstrating a lack of care for subordinate needs.

### HLBs and Microaggressions

Microaggression concerns a low-intensity workplace behavior that forms a conceptual neighbor of HLB Lack of Care as it relates to subtle everyday indirect uncaring behaviors. Racial microaggressions refer to the racial indignities, slights, mistreatment, or offenses that people of minority may face on a recurrent or consistent basis (Lui & Quezada, 2019; Sue et al., 2007). Authors have also focused on microaggressions toward members of other marginalized groups (e.g., those with a stigmatized health status, LGBTQ individuals). Microaggression refers to perceptions of subtle forms of discrimination that are not only perpetrated by leaders, but can also come from colleagues and subordinates. HLB Lack of Care more specifically refers to leader behavior that implies a lack of care about followers, regardless of their minority status.

## Toward Construct Validity and a Nomological Network of HLB

In addition to our model mapping the conceptual space of harmful leadership, we aim to develop a measure for the four HLBs. To start demonstrating construct validity we test whether our HLB scales relate with each other and with related constructs and diverge from unrelated constructs (see Table 2 for the predicted relationships with the variables we include in the five studies we undertook). As an indication of discriminant validity, HLB scales should be unrelated to dissimilar or non-overlapping constructs, such as personal characteristics of the rater such as age, gender or tenure (see e.g., Brown et al., 2005).

**Table 2:** Proposed relationships between harmful behavior leadership and important correlates

Construct	Prediction				Findings			
HLB	Excessive pressure	Intimidation	Self- centeredness	Lack of care	Excessive pressure	Intimidation	Self- centeredness	Lack of care
Discriminant validity personal characteristics								
Age	0	0	0	0	0	0	0	0
Gender	0	0	0	0	0	0	0	0
Hours worked per week	0	0	0	0	0	0	0	0
Contact with leader	0	0	0	0	0	0	0	0
Education	0	0	0	0	0	0	0	0
Tenure	0	0	0	0	0	0	0	0
Size of organization	0	0	0	0	0	0	0	0
Business sector	0	0	0	0	0	0	0	0
Nomological validity: leadership styles								
Abusive supervision*	+	+	+	+	+	+	+	+
DLB overall*	+	+	+	+	+	+	+	+
Ethical leadership	−	−	−	−	−	−	−	−
Initiating structure*	−	0	−	0	−	0	−	−
Consideration*	−	−	−	−	−	−	−	−
Laissez faire*	0	0	+	+	+	+	+	+
Management by exception active*	+	+	0	0	+	+	+	0
Management by exception passive*	0	0	+	+	+	+	+	+
Nomological validity: follower personality and attitudes								
Satisfaction with the leader	−	−	−	−	−	−	−	−
Engagement	−	−	−	−	−	−	−	−
Engagement dedication	−	−	−	−	−	−	−	−
Engagement absorption	−	−	−	−	−	0	−	−
Engagement vigor	−	−	−	−	−	0	−	−
Affective commitment	−	−	−	−	−	−	−	−
Deviance	+	+	+	+	+	+	+	+
Knowledge sharing	0	−	0	−	0	0	0	−
Knowledge hiding	+	+	+	+	+	+	+	0
Psychology safety	−	−	−	−	−	−	−	−
Perfectionism	+	0	0	0	+	0	0	0
Workaholism	+	0	0	0	+	0	0	0
Desire for control	+	+	0	0	+	0	0	0
Distrust	+	+	+	+	+	+	+	+
COVID-19	+	+	+	+	+	+	+	0

*Controlled for COVID-19 effects

### Proposed Relationships with Leadership Styles

We hypothesize that HLBs are positively related to other forms of unethical leadership and laissez faire behavior and negatively related to ethical leader behaviors, but also empirically distinguishable from them. We also expect HLB to be related to leader people and task orientation and passive and active management by exception (Bass & Bass, 2008).

Research suggests that different forms of abusive leadership (Keashley, 1998) are related to wider destructive leader behavior (Krasikova et al., 2013). As previously noted, destructive leadership behavior (Thoroughgood et al., 2012) depicts leaders acting in harmful ways. In the unethical leadership realm, abusive supervision is the most studied form of destructive leadership (Schyns & Schilling, 2013). We expect that all four harmful leader behavior dimensions will correlate positively with destructive leadership behavior and abusive supervision.

Hypothesis 1: All HLB dimensions correlate positively with abusive supervision (1a) and destructive leader behavior (1b).

Laissez faire leadership is a form of “non-leadership” (Bass & Bass, 2008) understood as a negative leadership style due to the undesired impact on outcomes. Thus, drawing on literature that suggests that less intense behaviors also cause harm (Neuman & Baron, 1998), we expect laissez faire leadership to be related to low-intensity HLBs.

Hypothesis 2: HLB Lack of Care and Self-Centeredness correlate positively with laissez faire leadership.

Ethical leadership is defined as “the process of influencing the activities of a group toward goal achievement in a socially responsible way” (De Hoogh & Den Hartog, 2009, p. 341). Ethical leaders are people-oriented and aim to be fair in the way they use their power, value the communication of ethical norms, and reinforce ethical behavior. HLB contrasts with ethical leadership as it involves injustice and harsh or uncaring treatment of subordinates, opposing the fair and respectful treatment involved in ethical leadership (Kalshoven & Den Hartog, 2013). Though ethical and harmful forms of leadership can be contrasted they are not poles of the same continuum (Einarsen et al., 2007). We expect the four HLBs to correlate negatively with perceived ethical leader behavior.

Hypothesis 3: HLB dimensions correlate negatively with ethical leadership.

As mentioned, though leaders have to focus both on people and goals, this basic distinction has not been considered in unethical leadership research yet. HLB Excessive Pressure for Results and Self-Centeredness are the task or goal-oriented forms of HLB in our model. As leaders high on initiating structure clearly define their own roles and those of their followers, we expect both goal-oriented HLB dimensions to be negatively correlated with leader initiating structure. Leaders high on consideration care about follower’s well-being, status, and contributions and are inclined to nurture rather than harm followers (Brown et al., 2005). Thus, we expect all HLBs to be negatively related to consideration.

Hypothesis 4: HLB Excessive Pressure for Results and Self Centeredness dimensions correlate negatively with leader initiating structure.

Hypothesis 5: HLB dimensions correlate negatively with leader consideration.

Leaders who actively manage by exception, “regularly monitor subordinates’ performance to see if the standards are being met. When they are more passive, they ask no more than the essential to get the job done” (Bass & Bass, 2008, p. 372). Active management by exception leaders take corrective action in anticipation and passive leaders only take action when problems arise (Tepper & Percy, 1994). We expect high-intensity HLBs to be positively related to active management by exception and the low-intensity HLBs to be positively related to passive management by exception.

Hypothesis 6: HLB Excessive Pressure for Results and Intimidation dimensions correlate positively with management by exception active.

Hypothesis 7: HLB Lack of Care and Self Centeredness dimensions correlate positively with management by exception passive.

### Proposed Relationships with Follower Personality, Attitudes and Behavior

Subordinates’ personality traits likely influence subjective evaluations of leaders’ harmful behaviors (Brees et al., 2016) as subordinates’ characteristics affect the attributions they make (Hackney & Perrewé, 2018). To date, only few studies have investigated subordinates personality in relation to unethical leader behavior (e.g., Big-5—Mackey et al., 2015; external attribution styles—Martinko et al., 2011; hostile attribution style and trait anger ––Brees et al., 2016). Here, we investigate relationships of perfectionism, workaholism, desire for control, and distrust of others with HLB perceptions.

Perfectionism is characterized by striving for flawlessness and setting of excessively high standards for performance accompanied by tendencies for overly critical self-evaluations and fear of negative evaluations by others (Stober & Otto, 2006). Perfectionists spend more time and effort at work than less perfectionistic individuals (Clark et al., 2016). Likewise, workaholism is defined as an uncontrollable need to work incessantly and involves working excessively, compulsively and harder than others (Clark et al., 2016; Schaufeli et al., 2008). We propose perfectionists and workaholics are likely to be more sensitive to task-oriented leader behaviors directed at goal achievement as results related thoughts are the main priority of such employees even when they are not at work (Widiger & Simonsen, 2005). As they pay more attention to goal cues, they may be more sensitive to performance pressure and quicker to interpret their leader’s behavior as Excessive Pressure.

Hypothesis 8: HLB Excessive Pressure for Results relates positively with (a) subordinates’ perfectionism and (b) workaholism.

Machiavellianism (Mach) is a trait that encompasses the inclination to “distrust others, engage in amoral manipulation, seek control over others, and seek status for oneself” (Dahling et al., 2009, p. 219). The negative outlook of those high on Mach makes them evaluate the behavior and intentions of others more negatively. Here, we address the relationship of two core Mach dimensions with HLB: desire for control and distrust of others. Those high on Mach believe others are threats and they attempt to control situations in order to dominate social settings. Subordinates that desire to control interpersonal situations and dilute others’ power are sensitive to signs of control loss. Consequently, they may more easily feel dominated and pressured and likely more quickly perceive the intense HLBs Intimidation and Excessive Pressure.

Hypothesis 9: High intensity HLB dimensions (Excessive Pressure for Results/Intimidation) relate positively with desire for control.

In addition, the Mach facet of distrust of others involves “a cynical outlook on the motivations and intentions of others with a concern for the negative implications that those intentions have for the self” (Dahling et al., 2009, p. 9). Hence, high Machs have a negative view of others including their leader and expect others to be untrustworthy (Christie, 1970), which likely makes them more prone to perceive leader behavior as harmful.

Hypothesis 10: HLB dimensions are positively correlated with distrust of others.

HLBs likely negatively relate to followers’ positive attitudes as subordinates working for unethical leaders have found to be less satisfied with their lives and job (e.g., Tepper, 2000), less engaged (Tepper et al., 2017) and less psychologically attached and committed (Schmid et al., 2019). Work engagement is defined as “a positive, fulfilling work-related state of mind characterized by vigor (…high levels of energy), dedication (…sense of significance), and absorption (…being fully concentrated)” (Schaufeli et al., 2002, p. 74). Ethical leaders facilitate work attachment, value employees and give meaning, which enhances positive attitudes. Unethical leadership presents contrary features (Barnes et al., 2015). We predict that HLB will be negatively related to employees’ satisfaction with the leader, employee engagement and affective commitment.

Hypothesis 11: Harmful leader behaviors correlate negatively with satisfaction with the leader (a), Engagement, that is Dedication (b), Vigor (c), Absorption (d) and affective commitment (e).

Unethical forms of leadership tend to positively relate to undesired outcomes such as workplace deviance (Schyns & Schilling, 2013; Tepper et al., 2017). Workplace deviance is defined as “voluntary behavior that violates significant organizational norms and in so doing threatens the well-being of an organization, its members, or both” (Robinson & Bennett, 1995, p. 556). Followers may feel inclined to retaliate harmful leader behavior and engage in deviance. In line with this, we expect harmful leader behavior to be positively related to work place deviance.

Hypothesis 12: Harmful leader behaviors correlate positively with deviance

Organizational performance increasingly relies on knowledge (Kim et al., 2016) and knowledge sharing is a desirable organizational outcome that can improve goal achievement (Wang et al., 2011). Knowledge sharing is defined as “the sharing of specialized knowledge, unique skills, expertise, and information” among employees in the organization (Lee et al., 2018, p. 403). Knowledge sharing demands that employees shift attention from their own work to share time and knowledge with coworkers, suggesting a care and concern for others. Research suggests that the extent to which employees feel they are taken care of predicts their willingness to share information (Kahn, 1993; Stiehl et al., 2018). Because HLBs display a lack of care for followers, we expect HLBs low on people orientation to be negatively related to knowledge sharing. In line with this, abusive supervision negatively affects the relationships among employees and increases distrust in teams (Tepper et al., 2017), which likely harms information exchange between team members. Abusive supervision was indeed shown to negatively relate to information exchange (Kim et al., 2016). We expect:

Hypothesis 13: HLB Intimidation and Lack of Care relate negatively with knowledge sharing.

In contrast, knowledge hiding is “an intentional attempt by an individual to withhold or conceal knowledge that has been requested by another person” (Connelly et al., 2012, p. 65). This is a counterproductive work behavior. Unethical forms of leadership tend to correlate positively with counterproductive behaviors (Schyns & Schilling, 2013). When subordinates experience HLBs, they may withhold knowledge from others as a way of seeking revenge. Although there is not yet much empirical work on this, one available study found that abusive supervision correlated positively with knowledge hiding (Khalid et al., 2018). Here, we expect this to also hold for the other HLBs and hypothesize:

Hypothesis 14: All HLBs relate positively with knowledge hiding.

Trusting their team and feeling comfortable in the workplace is vital to the quality of employees’ work life. Psychological safety is the belief that the workplace holds interpersonal trust and mutual respect which reassures people that they can take risks and be themselves (Edmondson, 1999). Leadership affects psychological safety (Newman et al., 2017) and HLB is expected to hurt psychological safety as such behaviors damage workplace relationships, employee well-being, and trust (Erickson et al., 2015; Xiaqi et al., 2012). We hypothesize:

Hypothesis 15: All HLB dimensions relate negatively to psychological safety.

## Measure Development

In developing the measure, we used the critical incident technique (Flanagan, 1954) to obtain specific behavioral examples of harmful leadership as basis for item generation (e.g., Thoroughgood et al., 2012). We wanted to develop an instrument that covered the four types of HLB and was parsimonious. Our measure was developed in five studies with unique samples. Study 1 was conducted to generate items and qualitatively confirm the hypothesized structure. Studies 2—4 were conducted to examine trait validity, internal coherence, nomological validity, measurement invariance, conduct power analysis and examine the incremental prediction of HLB. Study 5 aimed to confirm the multilevel fit of the structure and the use of hard data as an outcome at the team level with Bayesian analysis. With exception of Study 3 (measurement invariance with items in English, administered to native English speakers, mostly from the United Kingdom 86.8%), all items were administered in Portuguese in Brazil. The internal consistency estimates for the HLB scales were high in all studies (between 0.83 and 0.93).

### Study 1: Item Generation

Participants (35 employees—25 managers, for details about the sample used in this and subsequent studies, see Table 3) were asked to describe three situations in which a leader displayed harmful leader behavior, to describe the worst behavior a leader could display and to define harmful leader behavior. All interviews were performed in Brasilia, Brazil, in Portuguese. In total, 85 general critical incidents were obtained on which content and lexical analyses were performed. The coding process followed the prior formulated harmful leader behavior categories: (1) Intimidation, (2) Lack of Care, (3) Self-Centeredness, and (4) Excessive Pressure for Results. Coding rules were developed in order to specify the conditions under which a particular outlined behavior could be coded within a specific category (Crawford & Kelder, 2019). Five independent coders were instructed to perform the analysis. Additionally, content adequacy and validity of items, dimensions and the overarching construct were evaluated by an expert panel (five professors and two members of a leadership research group) beyond the research team (Crawford & Kelder, 2019). Finally, we generated 37 initial items representing the four dimensions.

**Table 3:** Summary of studies, their procedures, and data/sample characteristics

Study	Procedures and variables	Data/sample
Study 1	Critical incident technique = 85 critical incidents Reduction of initial pool to 37 items in four dimensions using independent coders and content analysis Expert rating of content adequacy Lexical analyses with software support (Iramuteq)	*N* = 35 employees—25 managers (snowball sampling/not hierarchically related) *M* age = 38.65 years 52% of the managers and 60% of subordinates were male different industries
Study 2	Item reduction CFA and reliability estimation Correlational analysis discriminant/nomological: ethical leadership, abusive supervision HLB and AS regressed on satisfaction with the leader Power analysis	*N* = 218 employees—snowball sampling *M* age = 37.09 years 42.2% male different industries
Study 3	CFA and reliability estimation Correlational analysis Discriminant/nomological: abusive supervision, destructive leadership, initiating structure and consideration, laissez faire, management by exception active and passive, engagement, affective commitment and deviance Measurement invariance Incremental prediction of HLB over employee attitudes	*N* = 352 employees *M* age = 35.89 years 54.8% female Panel study, Different industries
Study 4	CFA and reliability estimation Correlational analysis Discriminant/nomological: knowledge sharing, knowledge hiding, psychological safety, workaholism follower, perfectionism follower, desire for control follower, distrust	*N* = 160 employees Snowball sampling *M* age = 32.65 years 64% female different industries
Study 5	Multilevel CFA Bayesian analysis predicting absenteeism hard data	*N* = 1921 employees 196 units *M* age = 42 years 52.6% male

*AS*  abusive supervision

#### Lexical Analyses

In order to check if the same categories would emerge a posteriori from our data we performed lexical analysis with the support of the software Iramuteq (Ratinaud & Marchand, 2012) that analyzes words in a given context and classifies fragments in categories. Five categories were identified without researchers’ subjective interference. Four of them correspond to the ones hypothesized by HLB and had a homogenous percentage of content: Intimidation (13.2%), Excessive Pressure for Results (18.8%), Self-Centeredness (25.5%) and Lack of Care (25.5%). The last category focused on Control and consistency and encompassed mainly micromanaging behaviors and displaying tight control over the work process. These behaviors are not necessarily harmful, their effects depend on the workplace context (i.e., tightly controlling shifts might be destructive in a university, but part of the leader’s job description in a hospital). Therefore, the behaviors in this fifth category were not included in further studies.

### Study 2: A First Scale Validation

We administered the newly developed 37-item HLB questionnaire (see Table 4 for the items) to 218 employees (see Table 3 for sample details). *Ethical leadership* was assessed with 29 items from the Brazilian version (Almeida et al., 2018) of the Ethical Leadership at Work (ELW) scale (Kalshoven et al., 2011). An overall ethical leadership score was used. An example item is: My leader “Clarifies integrity guidelines”. In order to assess *Abusive Supervision*, a short form of Tepper’s (2000) scale with 6 items was used (Martinko et al., 2011; Mitchell & Ambrose, 2007). A sample item is: “My supervisor is rude to me”. The response scale for all leadership measures ranged from 1 (“never behaves in this way”) to 5 (“always behaves in this way”). *Satisfaction with the leader* was measured with 5 items developed by Siqueira (2008). A sample item is: “I am satisfied with the way my leader treats me”. The response scale ranged from 1 (“strongly disagree”) to 5 (“strongly agree”).

**Table 4:** Item and loadings from exploratory and confirmatory factor analyses per study

HLB dimensions and items	Study 2	Study 3	Study 4	Study 5			
	CFA	CFA	CFA	Multilevel CFA			
				Step 1	Step 5 within	Step 5 between	ICC
Self-centeredness (HLB SelfC)							
Shows favoritism to employees that contribute to his/her personal gain	0.85	0.79	0.80	0.80	0.79	0.96	0.08
Sabotages employees to self-promote	0.80	0.81	0.75	0.82	0.81	0.98	0.08
Puts personal interests above the people he/she works with	0.78	0.82	0.84	0.84	0.84	0.92	0.08
Takes credit for other people’s work as if he/she did it him/herself	0.76	0.85	0.72	0.53	0.51	0.72	0.05
Only rewards employees if and when they fulfill his/her personal wishes	0.59	0.82	0.74	0.61	0.60	0.80	0.06
Excessive pressure for results (HLB ExcP)							
Focus exclusively on results regardless of the team’s needs	0.83	0.85	0.79	0.80	0.77	0.96	0.12
Submits the team to a high level of stress to increase performance	0.82	0.80	0.85	0.81	0.78	0.99	0.17
Places excessive demands on employees	0.78	0.90	0.88	0.88	0.85	1	0.14
Overloads high performance employees	0.73	0.80	0.70	0.75	0.73	0.92	0.09
Regularly demands tasks that go beyond employees’ working hours	0.65	0.79	0.60	0.74	0.72	0.90	0.11
Pressures the team to finish tasks before the deadline	0.63	0.77	0.74	0.65	0.61	0.94	0.09
Intimidation (HLB Intim)							
Ridicules subordinates with low performance	0.85	0.92	0.80	0.81	0.78	0.99	0.11
Publically humiliates subordinates	0.84	0.91	0.88	0.88	0.85	1	0.15
Punishes behaviors that do not please him/her	0.80	0.85	0.86	0.66	0.65	0.86	0.06
Screams to get what he/she wants	0.72	0.81	0.71	0.82	0.80	0.95	0.14
Makes public threats to get what he/she wants*	0.60	0.82	0.79	–	–	–	–
Lack of care (HLB LackC)							
Is concerned with the well-being of employees (inverted)	0.87	0.75	0.83	0.89	0.87	0.96	0.13
Acknowledges efforts made by the employees (inverted)	0.81	0.83	0.85	0.88	0.87	0.98	0.08
Takes team’s demands into consideration (inverted)	0.79	0.73	0.84	0.78	0.77	0.99	0.07
Communicates in a transparent manner with the team (inverted)	0.78	0.65	0.83	0.84	0.82	0.97	0.11
Fit indexes							
							Multilevel
TLI	0.95	0.94	0.93	0.94	0.94	0.51	0.95**
CFI	0.95	0.95	0.94	0.95	0.95	0.58	0.95**
RMSEA	0.05	0.07	0.07	0.07	0.07	0.41	0.05**
SRMR	0.06	0.04	0.05	0.04	0.04	0.07	*W* = 0.04 *B* = 0.08**

*Item not included in Study 5

**Multilevel model fit indexes

We conducted a CFA (with ML estimation) using M-plus version 8 to confirm the HLB factor structure and to estimate goodness-of-fit (results in Table 4). We intended to develop an economical (i.e., not too long), yet reliable and valid measure. Therefore, further item selection was based on factor loadings, (see Henson & Roberts, 2006). The final instrument has 20 items (see Table 4). Fit indices for the four-dimensional model showed a good fit (CFI = 0.95, TLI = 0.95, RMSEA = 0.05, SRMR = 0.06). The internal consistency estimates for the HLBs were high (between 0.87 and 0.89).

The four factors solution explained a total of 68.15% of the variance, exceeding the minimum acceptable for scale development (Hinkin, 1998). The factor correlation matrix showed correlations above 0.38 between the proposed factors, implying that factors are related (Tabachnick & Fidell, 2007). The inter-correlations among the HLB scales range from *r* = 0.61 to 0.71 suggesting they are related but not identical. Descriptive statistics, correlations, and Cronbach’s α’s are displayed in Table 5.

**Table 5:** Study 2: Descriptive statistics, correlations, and Cronbach’s *α* for nomological and discriminant validity (*N* = 218)

Variables	*M*	*SD*	1	2	3	4	5	6	7	8	9
1. HLB ExcP	2.35	1.03	(0.88)								
2. HLB LackC	2.38	0.99	0.64**	(0.89)							
3. HLB Intim	1.75	0.93	0.68**	0.61**	(0.88)						
4. HLB SelfC	2.12	1.05	0.67**	0.69**	0.71**	(0.87)					
5. Abusive supervision	1.90	0.82	0.63**	0.62**	0.79**	0.69**	(0.84)				
6. Satisfaction with the leader	3.48	1.12	− 0.65**	− 0.85**	− 0.63**	− 0.75**	− 0.68**	(0.93)			
7. Ethical leadership	3.32	0.80	− 0.61**	− 0.89**	− 0.59**	− 0.73**	− 0.61**	0.83**	(0.95)		
8. Gender	–	–	0.07	0.10	0.09	0.11	0.07	− 0.09	− 0.06		
9. Tenure (years)	4.63	2	0.08	0.18**	0.07	0.11	0.00	− 0.17**	− 0.15*	− 0.07	

Cronbach’s alpha coefficients are displayed on the diagonal

**p < 0.01, *p < 0.05

Next, we began a multi-study effort to test the nomological net of HLB, starting in this study. Consistent with our predictions, all HLB dimensions correlated positively with abusive supervision (Hypothesis 1a) and negatively with ethical leadership (Hypothesis 3) and satisfaction with the leader (Hypothesis 11). As expected, HLB Intimidation correlated highest with abusive supervision (*r* = 0.79, *p* < 0.001). Surprisingly, HLB Lack of Care correlated highest with the ELW (*r* = − 0.89, *p* < 0.001) and satisfaction with the leader (*r* = − 0.85, *p* < 0.001). As expected, HLB dimensions were uncorrelated with employees’ gender and tenure, with the exception of Lack of Care that correlated weakly with tenure.

To further assess content validity, we also investigated the ability of HLBs to incrementally predict satisfaction with the leader beyond abusive supervision with a set of regressions (Table 6). In the first step, on its own, abusive supervision explained 45% of the variance in satisfaction with the leader (∆*R*^2^). The ∆*R*^2^ for step five (with abusive supervision and all HLB variables inserted) showed that HLB as a set explained an additional 32% of variance in satisfaction with the leader (Adj *R*^2^ = 0.77, *p* = 0.01) when compared with the first model having only abusive supervision as predictor (Adj *R*^2^ = 0.45, *p* = 0.01). More specifically, Lack of Care (*β* = − 0.58, *p* = 0.001), Self-Centeredness (*β* = − 0.23, *p* = 0.001) and abusive supervision (*β* = − 0.17, *p* = 0.001) account for a unique proportion of the variance even with all other dimensions present.

**Table 6:** Study 2: Summary of regression analyses (*N* = 218)

Variables	Satisfaction with the leader			
	Adj *R*^2^	∆*R*^2^	∆*F*	*β*
Step 1	0.45**	0.46**	180.53**	
Abusive supervision				− 0.68**
Step 2	0.47**	0.02**	9.86**	
Abusive supervision				− 0.48**
HLB intimidation				− 0.25**
Step 3	0.54**	0.06**	29.83**	
Abusive supervision				− 0.39**
HLB intimidation				− 0.08
HLB excessive pressure				− 0.35**
Step 4	0.62**	0.08**	48.17**	
Abusive supervision				− 0.26**
HLB intimidation				0.06
HLB excessive pressure				− 0.22**
HLB self-centeredness				− 0.46**
Step 5	0.77**	0.15**	142.97**	
Abusive supervision				− 0.17**
HLB intimidation				0.07
HLB excessive pressure				− 0.07
HLB self-centeredness				− 0.23**
HLB lack of care				− 0.58**
Step 6	0.76**	0.77**	175.78**	
HLB intimidation				− 0.02
HLB excessive pressure				− 0.08
HLB self-centeredness				− 0.26**
HLB lack of care				− 0.60**
Step 7	0.69**	0.69**	477.18**	
Overall HLB				− 0.83**

**p < 0.01

#### Power Analysis

**Fig. 2:**
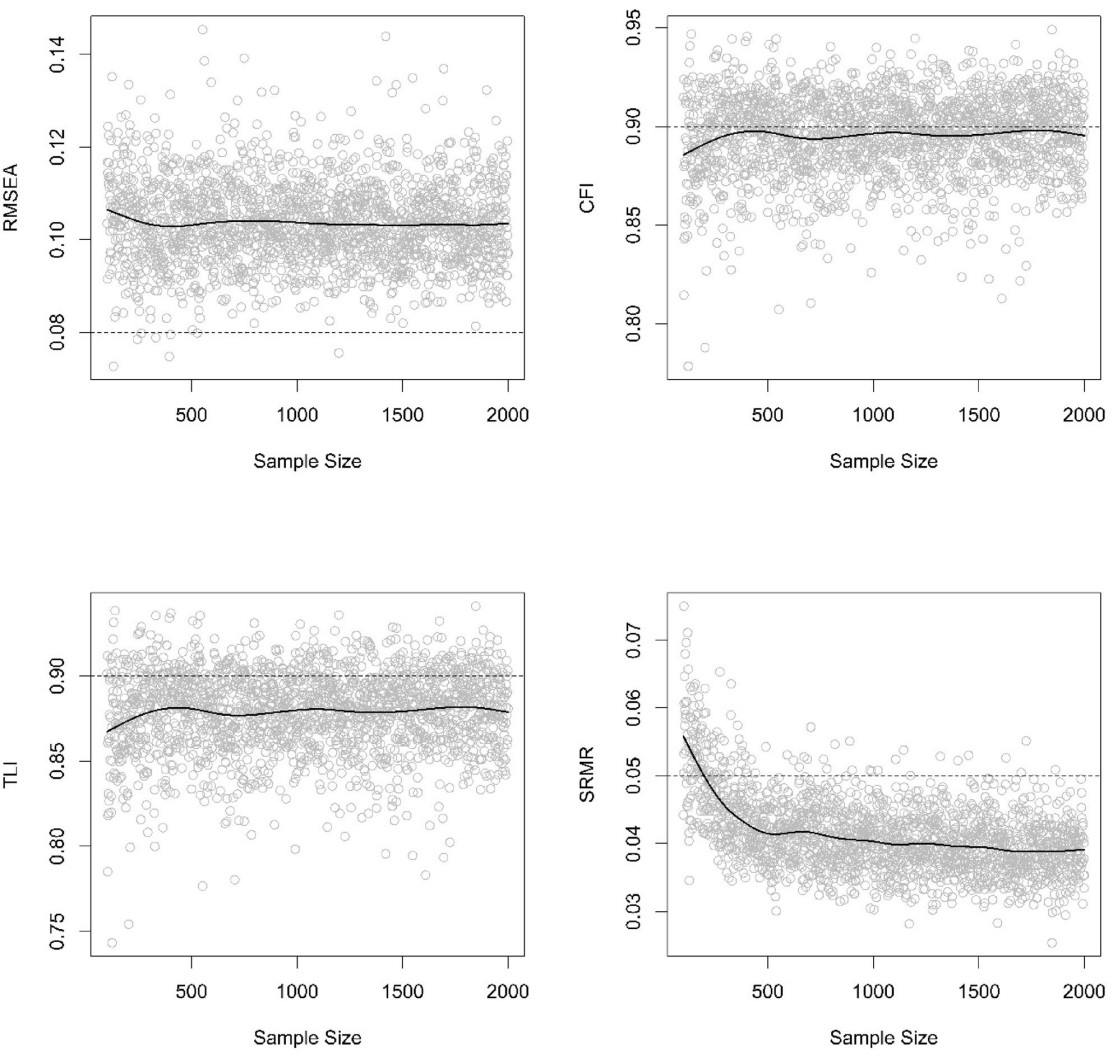
Power analysis RMSEA, CFI, TLI, and SRMR distribution from sample sizes from 100 to 2000 simulated participants

We used a Monte Carlo simulation study (Muthén & Muthén, 2002) to evaluate the necessary sample size to get good enough fit indices (i.e., RMSEA, CFI, TLI, SRMR). Figure 2 shows that in samples from 100 employees or more, apart from SRMR, the fit indexes have no clear turning point in which increasing sample size would improve the quality of the fit of the model (assuming the best model found in Study 2 as the true data generating process). For SRMR, sample sizes larger than 198 participants will more likely result in an appropriate fit for all the factor loadings, latent variable correlations, and estimated item variances.

### Study 3

In our third study, data were gathered in English. We conducted a CFA with maximum likelihood estimation. Based on the results from the power analysis of Study 2, we used data from a sample of 352 native English-speaking employees in a panel study. Fit indices were in line with recommended values and showed that the four-dimensional model also fit the data well when the instrument is used in English (CFI = 0.95, TLI = 0.94, RMSEA = 0.07, SRMR = 0.04). We also evaluated the ability of the HLB to incrementally predict relevant outcomes beyond abusive supervision with structural equation modeling and tested measurement invariance with the alignment under approximate invariance procedure (Muthén & Asparouhov, 2018). The idea behind this procedure is to estimate average factor loadings and item intercepts which maximize the likelihood of the data, given a threshold of difference acceptance between the compared groups. Using the value of 0.15 as the maximum allowed difference between the groups’ parameters, all items were found to be invariant.

In this study we also expanded the nomological validity of HLB (see Table 7 and 8). Destructive leadership behavior was assessed with 26 items from Thoroughgood et al. (2012). An overall score was used. An example item is: My leader “Is confrontational when interacting with subordinates”. In order to assess abusive supervision, Tepper’s (2000)15-item scale was used. A sample item is: “My supervisor is rude to me”. Initiating structure and consideration were assessed with the LBDQ-XII (Stogdill, 1963). “Encourages the use of uniform procedures” and “Is friendly and approachable” are sample items. We measured laissez faire, management by exception active and passive with 4 items each (Bass & Avolio, 2000). “Avoids getting involved when important issues arise”, “keeps track of all mistakes” and “demonstrates that problems must become chronic before I take action” are sample items.

**Table 7:** Study 3 -Descriptive statistics, correlations, and Cronbach’s *α* HLB and leadership nomological and discriminant validity (*N* = 352)

Variables	*M*	*SD*	1	2	3	4	5	6	7	8	9	10	11
1. HLB ExcP	3.78	2.06	(0.93)										
2. HLB LackC	4.01	1.88	0.40**	(0.83)									
3. HLB Intim	1.88	1.56	0.62**	0.34**	(0.93)								
4. HLB SelfC	2.74	1.89	0.70**	0.48**	0.74**	(0.91)							
5. AS	1.96	1.40	0.63**	0.44**	0.85**	0.80**	(0.96)						
6. DLB	1.55	0.71	0.62**	0.43**	0.80**	0.77**	0.85**	(0.96)					
7. Structure	6.79	1.28	− 0.15**	− 0.41**	− 0.07	− 0.23**	− 0.18**	− 0.23**	(0.89)				
8. Consideration	6.50	1.48	− 0.57**	− 0.72**	− 0.44**	− 0.66**	− 0.60**	− 0.55**	0.52**	(0.92)			
9. Laissez Faire	2.52	1.30	0.56**	0.52**	0.51**	0.67**	0.60**	0.65**	− 0.45**	− 0.67**	(0.90)		
10. Manag Exp Active	3.25	1.37	0.37**	0.09	0.45**	0.43**	0.41**	0.37**	0.13*	− 0.23**	0.27**	(0.85)	
11. Manag Exp Passive	2.97	1.35	0.55**	0.42**	0.50**	0.61**	0.57**	0.62**	− 0.37**	− 0.57**	0.73**	0.33**	(0.88)

Cronbach’s alpha coefficients are displayed on the diagonal

*AS* abusive supervision, *DLB* destructive leader behavior

**p < 0.01, *p < 0.05

Gender coded *1* female, *2* male

**Table 8:** Study 3: Descriptive statistics, correlations, and Cronbach’s *α* HLB and AS nomological and discriminant validity (*N* = 352)

Variables^1^	*M*	*SD*	1	2	3	4	5	6		7	8	9	10	11	12
1. HLB ExcP	3.78	2.06	(0.93)												
2. HLB LackC	4.01	1.88	0.40**	(0.83)											
3. HLB Intim	1.88	1.56	0.62**	0.34**	(0.93)										
4. HLB SelfC	2.74	1.89	0.70**	0.48**	0.74**	(0.91)									
5. AS	1.96	1.40	0.63**	0.44**	0.85**	0.80**	(0.96)								
6. Satisfaction	1.94	0.89	− 0.33**	− 0.42**	− 0.24**	− 0.35**	− 0.34**	(−)							
7. Engagement overall	4.48	1.17	− 0.23**	− 0.46**	− 0.13**	− 0.25**	− 0.23**	0.77**		(0.95)					
8. Engagement vigor	4.13	1.20	− 0.22**	− 0.42**	− 0.09	− 0.22**	− 0.19**	0.73**		0.93**	(0.90)				
9. Engagement dedication	4.69	1.35	− 0.27**	− 0.48**	− 0.17**	− 0.28**	− 0.27**	0.79**		0.95**	0.85**	(0.91)			
10. Engagement absorption	4.61	1.19	− 0.15**	− 0.38**	− 0.09	− 0.19**	− 0.17**	0.62**		0.92**	0.77**	0.80**	(0.81)		
11. Affective commitment	4.21	1.31	− 0.32**	− 0.42**	− 0.23**	− 0.34**	− 0.33**	0.76**		0.72**	0.67**	0.75**	0.60**	(0.89)	
12. Deviance	2.09	0.90	0.25**	0.23**	0.31**	0.30**	0.34**	− 0.31**		− 0.35**	− 0.30**	− 0.34**	− 0.33**	− 0.36**	(0.83)

Cronbach’s alpha coefficients are displayed on the diagonal

AS abusive supervision

**p < 0.01, *p < 0.05

^1^Controling for COVID-19

Gender coded *1* female, *2* male

*Employee satisfaction* was assessed with the item “How do you feel about the job you have now”? (Trevor, 2001). The response scale ranged from 1 (“dislike it very much”) to 4 (“like it very much”). To assess *engagement*, we used the Work Engagement Scale (Schaufeli & Bakker, 2004). Sample items are “At my work, I feel bursting with energy” (Vigor), “I am immersed in my work” (Absorption) and “I am proud on the work that I do” (Dedication). *Affective commitment* was measured with 8 items (Allen & Meyer, 1990), As a sample item we have “I enjoy discussing my organization with people outside it”. Finally, *workplace deviance* was investigated with 6 items (Tepper et al., 2008). Respondents were asked to report how often, for example, they are “wasting time on the job”. The response scale for the leadership and outcomes variables ranged from 1 (“never”) to 7 (“always”). As the study was done during the pandemic, as a control variable the impact of COVID-19 on the personal situation of the respondents was measured, using five items. Respondents indicated the extent to which COVID-19 had impacted their physical, mental and family health, and their financial and occupational status. The response scale ranged from 1 (“not at all”) to 5 (“a great deal”).

In line with our predictions, all HLB dimensions correlated positively with abusive supervision and destructive leadership (Hypotheses 1a and 1b). Similar to Study 2, HLB Intimidation showed the highest correlation with abusive supervision due to their overlapping content (*r* = 0.85 (Study 3) and *r* = 0.79 (Study 2), *p* < 0.001). In line with Hypothesis 2, HLB Lack of Care and Self-Centeredness correlated positively with laissez faire leadership (*r* = 0.52 and *r* = 0.67, *p* < 0.001). However, HLB Intimidation and Excessive pressure also did (*r* = 0.51 and *r* = 0.56, *p* < 0.001). Abusive supervision and destructive leadership correlated similarly with laissez faire (*r* = 0.60 and *r* = 0.65, *p* < 0.001). Both goal-oriented HLBs (Excessive Pressure and Self-Centeredness) correlated negatively with initiating structure (*r* = − 0.15 and *r* = − 0.23., *p* < 0.001), in line with Hypothesis 4. All HLB dimensions correlated negatively with consideration, in line with Hypothesis 5. We hypothesized that high-intensity HLBs (Excessive Pressure and Intimidation) would be positively related to active and low-intensity HLBs (Lack of Care and Self-Centeredness) would be positively related to passive management by exception (Hypotheses 6 and 7). HLB Excessive Pressure for Results and Intimidation indeed related positively to active management by exception (*r* = 0.37 and *r* = 0.45, *p* < 0.001). However, contrary to expectations, HLB Self-Centeredness also did (*r* = 0.43, *p* < 0.001). All HLB dimensions (not only Lack of Care and Self-Centeredness, Hypothesis 7) correlated positively with passive management by exception.

**Fig. 3:**
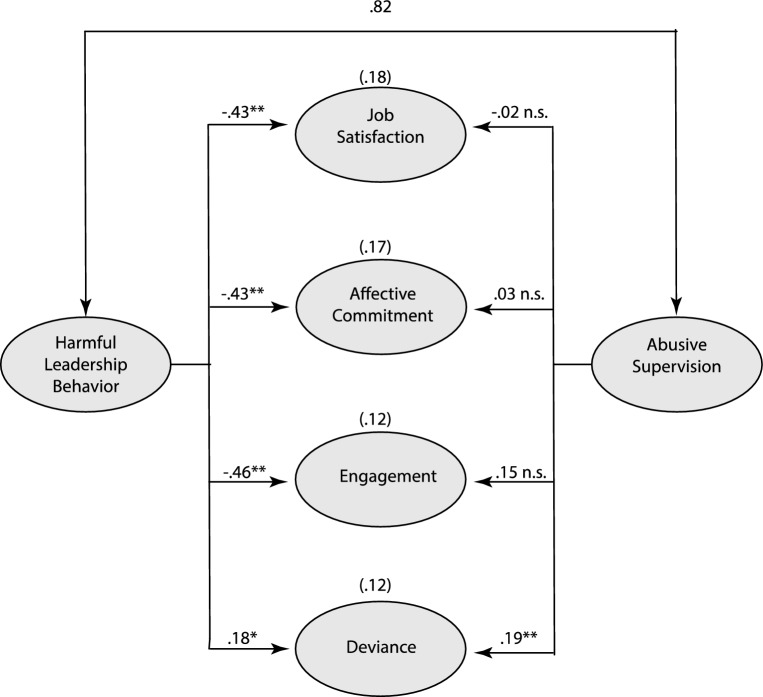
The simultaneous effects of harmful leadership and abusive supervision on outcomes. *Note* The numbers in parentheses are the proportions of explained variance **p < .01, *p < .05

All HLB dimensions related negatively to satisfaction and affective commitment and positively to deviance (Hypotheses 11a, 11e and 12). All HLB dimensions also related to all dimensions of work engagement (Hypotheses 11b, 11c and 11d) except for HLB Intimidation, this dimension only related to Dedication. Regarding incremental predictive power (estimated using M-plus version 8), when abusive supervision and overall HLB are predictors of employees’ attitudes and deviance in a structural equation model, abusive supervision is non-significant with the exception of predicting deviance, in which case both HLBs and abusive supervision are significant predictors (see Fig. 3).

### Study 4

We conducted a CFA using data from an independent sample of 160 employees (snowball sampling). This CFA also showed a good fit for the four-dimension HLB model (CFI = 0.94, TLI = 0.93, RMSEA = 0.07, SRMR = 0.05). Further investigating the nomological net, we administered the 20-item HLB measure (see Table 4). *Knowledge sharing* was measured with 5 items by Connelly et al. (2012). A sample item is: “told him/her exactly what s/he wanted to know”. *Knowledge hiding* was assessed with a 12-item measure (Connelly et al., 2012). A sample item is “said that I did not know, even though I did”. The response scale ranged from 1 (“totally disagree”) to 5 (“totally agree”). *Psychological safety* was assessed with a Brazilian 6-item version of Edmondson's (1999) measure. A sample item is “If you make a mistake on this team, it is often held against you”.

Trait *perfectionism* and *workaholism*, both 6-item scales, were measures from the computerized adaptive assessment of personality disorders—CATPD (Simms et al., 2011). Sample items are “I don’t consider a task finished until it's perfect” and “I am a workaholic, with little time for fun or pleasure”. Additionally, *desire for control* (3 items) *and distrust of others* (5 items) were measured with the Machiavellian personality scale (Dahling et al., 2009). Sample items are “I enjoy having control over other people” and “team members backstab each other all the time to get ahead”.

Results mostly support our hypotheses (Table 9). We expected that Intimidation and Lack of Care would be negatively correlated with knowledge sharing (Hypothesis 13). However, this hypothesis was only partially supported as only Lack of Care correlated negatively with knowledge sharing. Hypothesis 14 on knowledge hiding was partially supported as three HLB dimensions correlated negatively with it, but not Lack of Care. HLB variables also all correlated negatively with psychological safety, with slightly higher correlations for the people-oriented HLBs, largely supporting Hypothesis 15. The HLBs also correlated with subordinate personality traits. As expected, Excessive Pressure correlated positively with perfectionism and workaholism (in line with Hypothesis 8a and b). Partially supporting Hypothesis 9, desire for control related positively to Excessive Pressure, but not to Intimidation. As expected, all HLBs positively related to distrust of others (Hypothesis 10).

**Table 9:** Study 4: Descriptive statistics, correlations, and Cronbach’s α for nomological and discriminant validity (*N* = 160)

Variables	*M*	*SD*	1	2	3	4	5	6	7	8	9	10	11
1. HLB ExcP	2.62	1.04	(0.89)										
2. HLB LackC	2.36	1.06	0.58**	(0.90)									
3. HLB Intim	1.58	0.86	0.58**	0.54**	(0.90)								
4. HLB SelfC	2.11	1.06	0.70**	0.66**	0.68**	(0.88)							
5. K. Sharing	4.09	0.79	− 0.05	− 0.18*	− 0.10	− 0.11	(0.85)						
6. K. Hiding	1.59	0.64	0.28**	0.07	0.30**	0.27**	− 0.27**	(0.85)					
7. Psy safety	3.68	0.78	− 0.31**	− 0.48**	− 0.45**	− 0.41**	0.33**	− 0.20*	(0.76)				
8. Workaholism	3.06	0.89	0.28**	0.01	0.11	0.14	0.01	0.19*	− 0.09	(0.85)			
9. Perfectionism	3.18	0.88	0.28**	− 0.01	0.12	0.15	0.16*	0.11	0.01	0.66**	(0.87)		
10. D for control	2.65	0.90	0.19*	0.08	0.08	0.12	− 0.11	0.17*	− 0.07	0.31**	0.30**	(0.71)	
11. Distrust	2.37	0.85	0.30**	0.21**	0.19*	0.27**	− 0.07	0.26**	− 0.21**	0.20**	0.23**	0.34**	(0.81)

Cronbach’s alpha coefficients are displayed on the diagonal

**p < 0.01; *p < 0.05

Gender coded *1* female, *2* male

### Study 5

In our final study we investigated the multilevel structure of the HLB model and predicted hard absenteeism data at the team level using Bayesian analysis. Leadership research is frequently conducted with nested data as employees are clustered in workgroups and units and observations are likely to be correlated (Crawford & Kelder, 2019; Dyer et al., 2005). Multilevel CFA allowed us to estimate the effect of clusters.

Data were collected in an organization from the financial sector. The organization is spread all over Brazil with branches in all states. We were allowed to do a short employee survey and were provided unit level absenteeism data from the company’s archives. All 5,437 employees working at headquarters were invited to voluntarily participate in a university study. The survey was made available through the company intranet. The procedure and confidentiality, as well as the researcher responsible for data collection were presented to participants online. Participation was voluntary and in total, 1981 employees (from 256 units) agreed to participate. As all analyses were performed at the unit level, units with only one respondent (60 units) were excluded from the analyses. The final sample consists of 1,921 employees from 196 units (35,33%).

Data were collected at two different time points. At Time 1, employee’s perceptions of their leader’s behaviors were measured. At Time 2, 6 months later, hard data of unit absenteeism of the first semester of 2017 was collected. The time lag design made it possible to study the relation between leader behaviors and stress-related absenteeism in the period after survey data collection.

*Harmful leader behavior* was measured with the 20 HLB items (Study 2). However, one item (“Makes public threats to get what he/she wants”) showed a different pattern in this study, correlating negatively with the other items. This may be because of the setting (financial sector). Therefore, this item was removed from further analyses. The internal consistency estimates for the HLBs were also high (between 0.83 and 0.91). All items were rated on the aforementioned 5-point scale. Additionally, we measured *stress related absenteeism* using hard data from the organization. Health leaves were coded based on ICD (International Statistical Classification of Diseases and Related Health Problems) by the employee doctor and controlled by the organization’s HR department. For this study, we considered the stress related codes F40—F48 (anxiety, neurosis, stress-related and somatoform disorders, ICD-10, version 2016) as this type of absenteeism is likely more directly related to HLB factors than some other forms.

Stress-related absenteeism per unit was assessed by the organization with a function of the unit’s total number of days absent per the total number of days worked in the semester, consistent with the conceptual definition which states that absenteeism is “a lack of physical presence at a behavior setting when and where one is expected to be” (Harrison & Price, 2003, p. 204). Absenteeism presented a logistic distribution as many units do not have members absent for this reason in the study period. As the distribution of absenteeism was asymmetrical (Skewness = 4.05; Kurtosis = 17.09), we coded an index for the units using a dummy variable of units with (1) and without (0) absenteeism.

### Multilevel CFA

We followed the five steps proposed by Dyer et al. (2005) to perform multilevel CFA. We also aimed to estimate the effects of harmful leadership on absenteeism. To do this, we used an extension of the Bayesian hierarchical structural equation modeling procedure—BHSEM (Jiang & Mahadevan, 2009), where the two-parameter logistic model—2-PLM (Swaminathan & Gifford, 1985) was used as the measurement model (because our HLB measures are ordinal). For the hierarchical regression, we used a logistic model with an inverse logit link function. The second level estimates for HLB were modeled using the traditional Bayesian hierarchical model (Kruschke, 2015), which assumes scores from particular units come from a particular t-distribution with a mean of zero, allowing robust estimation of discrepant scores, resembling an additive model (Chan, 1998), but with correction for overdispersion.

Factor loadings were estimated for each item and an intercept and slope were estimated for the higher-level regression model using a Markov chain Monte Carlo—MCMC (Kruschke, 2015) method. We used non-informative priors (Kruschke, 2015) for the regression parameters and informative priors based on MLE estimates of the 2-PLM items’ difficulties and discriminations, which increase accuracy in complex models (Browne & Draper, 2000). We used the JAGS software (Plummer, 2003), interfaced with R through the R2jags package (Su & Yajima, 2012).

Regular CFA testing (Step 1 from Dyer et al., 2005) displayed an acceptable goodness-of-fit. Step 2 focuses on assessing between-group variation computing ICC(1). All HLB dimensions and items had nontrivial ICC values (Geldhof et al., 2014) indicating that there is a significant percentage of variance explained by group differences. ICC values for the items ranged from 0.05 to 0.17, with an average ICC of 0.10. For the factors, ICC values were: Intimidation = 0.12, Lack of Care = 0.11, Self-Centeredness = 0.09, and Excessive Pressure = 0.16. The within-group factor structure (Step 3) presented a goodness-of-fit similar to Step 1. However, the between-group factor structure (Step 4) on its own displayed a poor fit. Finally, MCFA (Step 5) was the best fitting model; suggesting a good fit at the within and an adequate one at the between level. Model fit, item ICCs and factor loadings and for Step 1 and 5 within and between are displayed in Table 4.

### Bayesian Analysis on the Relationship of Overall HLB with Absenteeism

Next, we conducted a Bayesian analysis to evaluate the overall effect of harmful leader behaviors on absenteeism. For estimation, we relied on the highest density interval (HDI; Kruschke, 2015), which shows the most credible values for the estimates, being the total probability inside the interval equal to some criterion, for instance, 95%. For hypothesis testing, we used a region of practical equivalence (Kruschke, 2015) combined with an overlapping coefficient (OVL; Inman & Bradley Jr, 1989). With this approach, one sets a distribution around a point of reference and then uses the OVL to see how much the estimate overlaps with this reference point. Here we set the point of reference with the same distribution as our estimates (i.e., normal distributions) with means equal to zero and standard deviation equals to the standard deviation of each of the estimates.

We found that both the slope and the intercept have positive means and HDIs; 1.41, 95% HDI [0.39; 2.82] and 0.83, 95% HDI [0.22; 1.60], respectively. This is evidence for a positive relation between the overall harmful leader behaviors and absenteeism. Interpreted in percentages, the value of the slope indicates that units with an average level of harmful leader behavior have an average probability of stress-related absenteeism of 69.6%. For further evaluation of the model, we also have found that the OVL for the slope is equal to 26.3 and 24.3% for the intercept. This means that our results show that units with higher levels of harmful leader behavior also have more stress-related absenteeism as the average harmful leader behavior level is enough to generate a substantial probability of absenteeism (69.9%). Also, the positive slope shows that increasing one standard deviation of harmful leader behavior results in a meaningful increase in the probability of stress-related absenteeism. The low value of OVL indicates that our estimates are quite robust, with minimal overlap with null-effects.

## Discussion

Over the past 20 years, unethical forms of leadership that are harmful to subordinates have received increasing research attention. Despite scholars arguing that such leadership is a complex and multi-faceted phenomenon (e.g., Schyns & Schilling, 2013), research to date has predominantly focused on abusive supervision and less on other harmful behaviors. Here, we developed a model of four types of HLB varying in their intensity and in their focus on either people or tasks/goals (Intimidation, Lack of Care, Self-Centeredness and Excessive Pressure for Results) and we mapped out the conceptual space around these. We also developed a measure, tested its psychometric properties and provide initial validity evidence for these four new scales in five studies.

The four HLB dimensions relate in expected ways to other constructive (ethical leadership) and destructive (abusive supervision and destructive leadership behavior) leadership variables (Studies 2 and 3). Also, in Study 3, HLB task/goal orientation dimensions were negatively related to initiating structure and all HLB dimensions were negatively related to leader consideration. However, contrary to what was expected, HLB Lack of Care was also negatively correlated with initiating structure.

Additionally, as expected, HLB Intimidation and Excessive Pressure were positively related to active management by exception. Yet, Self-Centeredness also was positively related to active management by exception, signaling that it’s indeed perceived as less intense but not as a passive form of leader behavior. While HLB Lack of Care and Self-Centeredness were indeed positively correlated with passive management by exception, HLB Excessive Pressure, and Intimidation also were. Perhaps the use of corrective action when problems arise, which is part of passive management by exception, stirs impressions of a more active form of management. Also, though we expected HLB Self-Centeredness and Lack of Care to be the only ones related to laissez faire, all unethical leadership variables were found positively related to laissez faire leadership (HLB dimensions, abusive supervision, and destructive leadership behavior). Future research on the link between harmful leader behavior and more passive forms of leadership would thus be of interest to study.

In addition, we showed how HLB dimensions relate negatively to desired correlates or outcomes (Study 2: satisfaction with the leader; Study 3: engagement, satisfaction, and affective commitment; Study 4: knowledge sharing and psychological safety), and positively to undesired correlates or outcomes (Study 3: deviance, Study 4: knowledge hiding; Study 5: stress-related absenteeism). Findings were mostly in line with expectations. For engagement, contrary to expectations, HLB Intimidation was only related to Dedication, meaning that high-intensity interpersonal abuse seems to mostly harm a sense of significance and enthusiasm about one’s work. Future studies should investigate this further.

Only very few studies to date relate harmful leadership with hard outcome data and Tepper et al. (2017) especially emphasized the need to evaluate whether abusive leaders affect absenteeism. In Study 5, we assessed how HLB relates to absenteeism with objective unit-level data provided by the organization on stress-related health leaves, answering this call as well as the call to investigate correlates of this type of leadership with different data sources (Hackney & Perrewé, 2018). Our data suggest that units with more perceived HLB also tend to have more stress-related absenteeism. Future studies can also address other forms of absenteeism as well as other hard measures HLB might affect.

We showed that HLB perceptions relate to subordinate traits, suggesting some people may be more prone to interpreting the behavior of their leader as harmful than others. As hypothesized, trait workaholism and perfectionism were positively related to perceived Excessive Pressure for Results. Mach is also associated with how individuals interpret the behavior of others as Machiavellians typically have a negative world view and a dim view of others and their intentions. Our findings show that those high on distrust of others perceive more HLB. Those high in Mach don’t trust others in general (Dahling et al., 2009) and they also seem to distrust their leader, thus likely more easily assuming negative intentions on the part of their leader and likely more quickly perceiving their behavior as harmful. These findings suggests that studies investigating harmful leader behaviors should not only follow a leader centric approach but also further address contextual variables and employees characteristics as they may have affect perceptions of or have an interactive effect with such behaviors (Tepper et al., 2001).

Also, some traits may make it more likely for subordinates to become the target of harmful behavior. For example, workaholics can actively create more work for themselves (Schaufeli et al., 2008) and perfectionists can show high quality performance (Stober & Otto, 2006). It is possible that leaders who are high on Excessive Pressure for Results will notice these tendencies and actively demand even more (cf. Widiger & Simonsen, 2005) from employees high on workaholism and perfectionism as they are already task oriented and driven to work hard. Thus, such traits may either make individuals sensitive to performance cues or may make them more likely to be targeted with excessive pressure (or both). Both the role of subordinate traits in leadership perceptions as well as the role of traits in becoming the target of harmful behavior need more research attention.

Future work could also investigate organizations as “bad barrels” (Kish-Gephart et al., 2010) as certain harmful behaviors (e.g., Excessive Pressure) may foster bottom-line results despite being harmful to subordinates. If that is the case, higher-level managers may condone or even reward such behavior from their lower-level line managers. Also, the literature shows that intimidation can be used as a way to punish low achievers (Ferris et al., 2007). However, this may come at the cost of employee well-being and absenteeism, thus is not only unethical but also potentially costly.

Taken together, our results support the differentiation between the four types of HLB proposed as they relate similarly to some yet differentially to other variables. In study 5, we also addressed the use of nested data in leadership and organizational studies (Dyer et al., 2005). As employees are nested in teams/units and other clusters, analyses should take the influence of higher levels into account when multiple respondents come from the same unit (Stapleton et al., 2016). The same is true for construct validation and CFA (Dyer et al., 2005; Stapleton et al., 2016). In line with the literature, our findings support the multilevel CFA as the best fitting solution for the nested data of Study 5. The literature has called for investigations of destructive forms of leadership at different levels (Hackney & Perrewé, 2018). The HLB measure can be used in a multilevel manner and we show initial evidence that HLB at the team-level impacts an important work outcome (stress related absenteeism).

Usually, studies of harmful leadership focus on a single form of such leadership. The HLB model and measure developed here aim to provide a more differentiated, yet still parsimonious alternative to study several potentially harmful forms of leadership at the same time. While the unidimensional measure for abusive supervision (Tepper, 2000) constitutes an excellent alternative when interested in better understanding high-intensity destructive leader behaviors (e.g., public outbursts), the literature has repeatedly suggested that models should take other forms of harmful behavior into account and encompass both high and low-intensity forms of harmful behavior (e.g., Thoroughgood et al., 2012). Given that some of these forms may be less visible, they may more easily be tolerated by organizations and might thus be more prevalent. While this idea is in need of further research, the fact that in all of our studies the mean score on Intimidation was lower than that of the other HLBs does suggest these other forms are more often experienced. Future work can also address how frequently the different HLB types occur in different settings.

The four dimensions we proposed correlate suggesting that leaders might indeed (be perceived to) behave in more than one harmful and unethical way. The proposed measure has several empirical and theoretical advantages over a unidimensional scale. For example, the separate dimensions may help to further explain the different processes by which harmful leadership affects employees and organizations and to better understand the relationships with different outcomes, which would be of interest to explore in future work. As a practical implication, identifying harmful behaviors and differences among them can contribute to the development of more specific (and accurate) organizational policies to fight such behaviors (Keashley, 1998) and provide better tools and interventions to organizations to counteract them (Hackney & Perrewé, 2018).

### Limitations and Further Suggestions for Future Research

The current research also has limitations. As our data are cross-sectional, it’s not possible to infer causality. In Study 5, we were able to gather multisource data at two time points, but in Study 2, 3, and 4 as our initial validation studies we used self-report measures and data collection took place at one point in time. In order to minimize common source variance, we informed the participants about the anonymity of participation. We suggest subsequent research to utilize multiple sources whenever possible to avoid this potential bias. Additionally, future research can address leaders’ harmful behaviors with longitudinal designs to address causality and to better understand how patterns of (perceptions of) such behavior develop over time (Hackney & Perrewé, 2018).

In Study 2 and 4, data were collected from convenience samples of workers through a snowball procedure and no response rate could be calculated. The advantage of the approach was that we could collect anonymous data from employees from many different settings and sectors and the findings and factor structure remained quite stable across the different samples. Moreover, as establishing construct validity is a continuous process, future studies should include additional correlates to test a wider nomological network for HLBs. Specifically, we encourage researchers in future studies to examine the differences in the nomological networks among the different HLB types to help further assess the usefulness and necessity of parsing out these types.

Also, while the literature converges on the fact that harmful behaviors still occur in workplaces, why this is the case is unclear. These forms of leadership are mostly associated with undesired outcomes (Tepper et al., 2017), thus one expects organizations would want to minimize them. However, such leadership may at times be maintained through the support of leaders or coworkers (Hackney & Perrewé, 2018) and this must be better understood. As noted, the low-intensity behaviors especially might be more tolerated as they are harder to perceive and make sense of (Neuman & Baron, 1998). Additionally, Excessive Pressure for Results fosters performance over other priorities, yet using excessive and harmful means. Such excessive pressure may at times be supported (and even applauded) from the top as leaders outstanding goal achievements can be reinforced even when they do it at the expense of others (Ma et al., 2004), allowing this type of harmful leadership to flourish. More work on this is needed.

Finally, future research can also investigate the role of moderators (e.g., ethical climate) that may influence the relation between perceptions of harmful leadership and antecedents or outcomes (e.g., Kalshoven et al., 2011). Such work might for example, test whether certain forms of organizational control can lower the occurrence of harmful behavior. In general, studying harmful leader behaviors and when and why they occur is important as it helps further our understanding of unethical leadership processes and develop ideas on how organizations might prevent or keep problems with harmful leadership in check.

## ^1^Note

The following items were not considered in further analysis: Intimidation (7 items): “Ridicules questions made bqwdsy employees to discourage questioning”, “Embarrasses the employee when not satisfied by the work”, “Expresses him/herself constantly in an aggressive manner when contradicted”, “Outsources unpleasant tasks to punish subordinates”, “Harms employees that contradict him/her”, “Punishes employees with nonsense tasks, “Uses the silent treatment to punish employees”, Lack of Care (3 items): “Considers the employee’s needs”, “Accepts negative feedback from subordinates even when it affects his/her image”, “Takes responsibility for mistakes even if they compromise his/her image”, Self-Centeredness (4 items): “Retains work-related information to remain in power”, “Takes responsibility for team mistakes even when it harms his/her image”, “Blames the team for personal dissatisfactions”, “Undervalues the team’s work to preserve personal power”, Excessive Pressure for Results (3 items): “Demands greater performance from the team than necessary to achieve excellent results”, “Imposes targets that exceed the capacity of the employees”, “Manipulates information to reach results”.

## References

[CR1] Allen NJ, Meyer JP (1990). The measurement and antecedents of affective, continuance and normative commitment to the organization. Journal of Occupational Psychology.

[CR2] Almeida J, Den Hartog DN, Porto JB (2018). Escala de liderança ética no trabalho: Evidências de validade da versão brasileira. Revista Psicologia Organizações e Trabalho.

[CR3] Ashforth B (1994). Petty tyranny in organizations. Human Relations.

[CR4] Babalola MT, Greenbaum RL, Amarnani RK, Shoss MK, Deng Y, Garba OA, Guo L (2020). A business frame perspective on why perceptions of top management’s bottom-line mentality result in employees’ good and bad behaviors. Personnel Psychology.

[CR5] Barling J, Christie A, Turner N (2008). Pseudo-transformational leadership: Towards the development and test of a model. Journal of Business Ethics.

[CR6] Barnes CM, Lucianetti L, Bhave DP, Christian MS (2015). You wouldn’t like me when I’m sleepy: Leaders’ sleep, daily abusive supervision, and work unit engagement. Academy of Management Journal.

[CR7] Bass BM, Avolio BJ (2000). MLQ multifactor leadership questionnaire.

[CR8] Bass BM, Bass R (2008). The Bass handbook of leadership. Theory, research, and managerial applications.

[CR9] Bernard M, Avolio BJ (1997). Full range leadership development: Manual for the multifactor leadership questionnaire. APA psyc tests.

[CR10] Brees J, Martinko M, Harvey P (2016). Abusive supervision: Subordinate personality or supervisor behavior. Journal of Managerial Psychology.

[CR11] Brown ME, Treviño LK, Harrison DA (2005). Ethical leadership: A social learning perspective for construct development and testing. Organizational Behavior and Human Decision Processes.

[CR12] Browne WJ, Draper D (2000). Implementation and performance issues in the Bayesian and likelihood fitting of multilevel models. Computational Statistics.

[CR13] Buch R, Martinsen ØL, Kuvaas B (2015). The destructiveness of laissez-faire leadership behavior: The mediating role of economic leader–member exchange relationships. Journal of Leadership and Organizational Studies.

[CR14] Chan D (1998). Functional relations among constructs in the same content domain at different levels of analysis: A typology of composition models. Journal of Applied Psychology.

[CR15] Christie R (1970). Studies in machiavellianism.

[CR16] Christie A, Barling J, Turner N (2011). Pseudo-transformational leadership: Model specification and outcomes 1. Journal of Applied Social Psychology.

[CR17] Clark MA, Michel JS, Baltes BB (2016). All work and no play? A meta-analytic examination of the correlates and outcomes of workaholism. Journal of Management.

[CR18] Connelly CE, Zweig D, Webster J, Trougakos J (2012). Knowledge hiding in organizations. Journal of Organizational Behavior.

[CR19] Cortina LM, Magley VJ, Williams JH, Langhout RDL (2001). Incivility in the workplace: Incidence and impact. Journal of Occupational Health Psychology.

[CR20] Cramwinckel FM, De Cremer D, van Dijke M (2013). Dirty hands make dirty leaders? The effects of touching dirty objects on rewarding unethical subordinates as a function of a leader’s self-interest. Journal of Business Ethics.

[CR21] Crawford JA, Kelder J (2019). Do we measure leadership effectively? Articulating and evaluating scale development psychometrics for best practice. The Leadership Quarterly.

[CR22] Dahling JJ, Whitaker BG, Levy PE (2009). The development and validation of a new Machiavellianism Scale. Journal of Management.

[CR23] De Hoogh AHB, Den Hartog DN, Tjosvold D, Wisse B (2009). Ethical leadership: The socially responsible use of power. Power and interdependence in organizations.

[CR24] Dyer NG, Hanges PJ, Hall RJ (2005). Applying multilevel confirmatory factor analysis techniques to the study of leadership. The Leadership Quarterly.

[CR25] Edmondson A (1999). Psychological safety and learning behavior in work teams. Administrative Science Quarterly.

[CR26] Einarsen S, Aasland MS, Skogstad A (2007). Destructive leadership behaviour: A definition and conceptual model. Leadership Quarterly.

[CR27] Einarsen S, Hoel H, Notelaers G (2009). Measuring exposure to bullying and harassment at work: Validity, factor structure and psychometric properties of the negative acts questionnaire-revised. Work and Stress.

[CR28] Erickson A, Shaw B, Murray J, Branch S (2015). Destructive leadership: Causes, consequences, and countermeasures. Organizational Dynamics.

[CR29] Ferris GR, Zinko R, Brouer RL, Buckley MR, Harvey MG (2007). Strategic bullying as a supplementary, balanced perspective on destructive leadership. Leadership Quarterly.

[CR30] Fisher, B. M., & Edwards, J. E. (1988). Consideration and initiating structure and their relationships with leader effectiveness: A meta-analysis. *Best Paper Proceedings, Academy of Management*, 201–205.

[CR31] Flanagan JC (1954). The critical incident technique. Psychological Bulletin.

[CR32] Flores GL, Posthuma RA, Campion MA, Ashkanasy NM, Bennett RJ, Martinko MJ (2016). Managing the risk of negative effects of high performance work practices. Understanding the high performance workplace. The line between motivation and abuse.

[CR33] Geldhof GJ, Preacher KJ, Zyphur MJ (2014). Reliability estimation in a multilevel confirmatory factor analysis framework. Psychological Methods.

[CR34] Greenbaum RL, Mawritz MB, Eissa G (2012). Bottom-line mentality as an antecedent of social undermining and the moderating roles of core self-evaluations and conscientiousness. Journal of Applied Psychology.

[CR35] Hackney KJ, Perrewé PL (2018). A review of abusive behaviors at work: The development of a process model for studying abuse. Organizational Psychology Review.

[CR36] Harrison DA, Price KH (2003). Context and consistency in absenteeism: Studying social and dispositional influences across multiple settings. Human Resource Management Review.

[CR37] Henson RK, Roberts JK (2006). Use of exploratory factor analysis in published research: Common errors and some comment on improved practice. Educational and Psychological Measurement.

[CR38] Hinkin TR (1998). A brief tutorial on the development of measures for use in survey questionnaires. Organizational Research Methods.

[CR39] Hogan R, Kaiser RB (2005). What we know about leadership. Review of General Psychology.

[CR40] House RJ, Howell JM (1992). Personality and charismatic leadership. The Leadership Quarterly.

[CR41] Inman HF, Bradley EL (1989). The overlapping coefficient as a measure of agreement between probability distributions and point estimation of the overlap of two normal densities. Communications in Statistics-Theory and Methods.

[CR42] Jiang X, Mahadevan S (2009). Bayesian structural equation modeling method for hierarchical model validation. Reliability Engineering & System Safety.

[CR43] Jonason PK, Webster GD (2010). The dirty dozen: A concise measure of the dark triad. Psychological Assessment.

[CR44] Judge TA, Piccolo RF, Ilies R (2004). The forgotten ones? The validity of consideration and initiating structure in leadership research. Journal of Applied Psychology.

[CR45] Kahn WA (1993). Caring for the caregivers: Patterns of organizational caregiving. Administrative Science Quarterly.

[CR46] Kalshoven K, Den Hartog D (2013). Ethical and unethical leader behaviors and their impact on individual well-being and deviance. Handbook of unethical work behavior: Implications for individual well-being.

[CR47] Kalshoven K, Den Hartog DN, De Hoogh AHB (2011). Ethical leadership at work questionnaire (ELW): Development and validation of a multidimensional measure. Leadership Quarterly.

[CR48] Kaplan RE, Kaiser RB (2003). Developing versatile leadership. MIT Sloan Management Review.

[CR49] Keashley L (1998). Emotional abuse in the workplace: Conceptual and empirical issues. Journal of Emotional Abuse.

[CR50] Khalid M, Bashir S, Khan AK, Abbas N (2018). When and how abusive supervision leads to knowledge hiding behaviors: An Islamic work ethics perspective. Leadership & Organization Development Journal.

[CR51] Kiazad K, Restubog SLD, Zagenczyk TJ, Kiewitz C, Tang RL (2010). In pursuit of power: The role of authoritarian leadership in the relationship between supervisors’ Machiavellianism and subordinates’ perceptions of abusive supervisory behavior. Journal of Research in Personality.

[CR52] Kim SL, Lee S, Yun S (2016). Abusive supervision, knowledge sharing, and individual factors. Journal of Managerial Psychology.

[CR53] Kish-Gephart JJ, Harrison DA, Treviño LK (2010). Bad apples, bad cases, and bad barrels: Meta-analytic evidence about sources of unethical decisions at work. The Journal of Applied Psychology.

[CR54] Krasikova DV, Green SG, LeBreton JM (2013). Destructive leadership: A theoretical review, integration, and future research agenda. Journal of Management.

[CR55] Kruschke JK (2015). Doing Bayesian data analysis: A tutorial with R, JAGS, and Stan.

[CR56] Lambert LS, Tepper BJ, Carr JC, Holt DT, Barelka AJ (2012). Forgotten but not gone: An examination of fit between leader consideration and initiating structure needed and received. Journal of Applied Psychology.

[CR57] Lee S, Loretta S, Yun S (2018). A moderated mediation model of the relationship between abusive supervision and knowledge sharing. The Leadership Quarterly.

[CR58] Lui PP, Quezada L (2019). Associations between microaggression and adjustment outcomes: A meta-analytic and narrative review. Psychological Bulletin.

[CR59] Ma H, Karri R, Chittipeddi K (2004). The paradox of managerial tyranny. Business Horizons.

[CR60] Mackey JD, Frieder RE, Perrewé PL, Gallagher VC, Brymer RA (2015). Empowered employees as social deviants: The role of abusive supervision. Journal of Business and Psychology.

[CR61] Maner JK, Mead NL (2010). The essential tension between leadership and power: When leaders sacrifice group goals for the sake of self-interest. Journal of Personality and Social Psychology.

[CR62] Martinko MJ, Harvey P, Sikora D, Douglas SC (2011). Perceptions of abusive supervision: The role of subordinates’ attribution styles. Leadership Quarterly.

[CR63] Meuser JD, Gardner WL, Dinh JE, Hu J, Liden RC, Lord RG (2016). A network analysis of leadership theory: The infancy of integration. Journal of Management.

[CR64] Mitchell MS, Ambrose ML (2007). Abusive supervision and workplace deviance and the moderating effects of negative reciprocity beliefs. Journal of Applied Psychology.

[CR65] Molero F, Recio P, García-Ael C, Fuster MJ, Sanjuán P (2013). Measuring dimensions of perceived discrimination in five stigmatized groups. Social Indicators Research.

[CR66] Muthén B, Asparouhov T (2018). Recent methods for the study of measurement invariance with many groups: Alignment and random effects. Sociological Methods and Research.

[CR67] Muthén LK, Muthén BO (2002). How to use a Monte Carlo study to decide on sample size and determine power. Structural Equation Modeling.

[CR68] Neuman JH, Baron RA (1998). Workplace violence and workplace aggression: Evidence concerning specific forms, potential causes, and preferred targets. Journal of Management.

[CR69] Nevicka B, De Hoogh AH, Den Hartog DN, Belschak FD (2018). Narcissistic leaders and their victims: Followers low on self-esteem and low on core self-evaluations suffer most. Frontiers in Psychology.

[CR70] Newman A, Donohue R, Eva N (2017). Psychological safety: A systematic review of the literature. Human Resource Management Review.

[CR71] Niven K, Healy C (2016). Susceptibility to the ‘dark side’ of goal-setting: Does moral justification influence the effect of goals on unethical behavior?. Journal of Business Ethics.

[CR72] Plummer, M. (2003). JAGS: A program for analysis of Bayesian graphical models using Gibbs sampling. In *Proceedings of the 3rd international workshop on distributed statistical computing*

[CR73] Ratinaud, P., & Marchand, P. (2012). Application de la méthode ALCESTE à de “gros” corpus et stabilité des “mondes lexicaux”: analyse du “CableGate” avec IRaMuTeQ. *Actes des 11eme Journées internationales d’Analyse statistique des Données Textuelles*, 835–844.

[CR74] Robinson SL, Bennett RJ (1995). A typology of deviant workplace behaviors: A multidimensional scaling study. Academy of Management Journal.

[CR75] Schaufeli WB, Bakker AB (2004). Job demands, job resources, and their relationship with burnout and engagement: A multi-sample study. Journal of Organizational Behavior.

[CR76] Schaufeli WB, Salanova M, Gonzalez-Romá V, Bakker AB (2002). The measurement of engagement and burnout: A confirmative analytic approach. Journal of Happiness Studies.

[CR77] Schaufeli WB, Taris TW, Van Rhenen W (2008). Workaholism, burnout, and work engagement: Three of a kind or three different kinds of employee well-being?. Applied Psychology.

[CR78] Schmid EA, Pircher Verdorfer A, Peus CV (2019). Shedding light on leaders’ self-interest: Theory and measurement of exploitative leadership. Journal of Management.

[CR79] Schmidt, A. A. (2008).* Development and validation of the toxic leadership scale* (Doctoral dissertation)

[CR80] Schyns B, Schilling J (2013). How bad are the effects of bad leaders? A meta-analysis of destructive leadership and its outcomes. Leadership Quarterly.

[CR81] Simms LJ, Goldberg LR, Roberts JE, Watson D, Welte J, Rotterman JH (2011). Computerized adaptive assessment of personality disorder: Introducing the CAT-PD project. Journal of Personality Assessment.

[CR82] Siqueira MMM, Siqueira MMM (2008). Satisfação no trabalho. Medidas do comportamento organizacional Ferramentas de diagnóstico e de gestão.

[CR83] Skogstad A, Einarsen S, Torsheim T, Aasland MS, Hetland H (2007). The destructiveness of laissez-faire leadership behavior. Journal of Occupational and Health Psychology.

[CR84] Stapleton LM, Yang JS, Hancock GR (2016). Construct meaning in multilevel settings. Journal of Educational and Behavioral Statistics.

[CR85] Stiehl E, Kossek EE, Leana C, Keller Q (2018). A multilevel model of care flow: Examining the generation and spread of care in organizations. Organizational Psychology Review.

[CR86] Stober J, Otto K (2006). Positive conceptions of perfectionism. Personality and Social Psychology Review.

[CR87] Stogdill RM (1963). Manual for the leader behaviour description questionnaire—form XII. An experimental revision.

[CR88] Su, Y. S., & Yajima, M. (2012). R2jags: A package for running jags from R. Retrieved from https://cran.r-project.org/web/packages/R2jags/R2jags.pdf

[CR89] Sue DW, Capodilupo CM, Torino GC, Bucceri JM, Holder A, Nadal KL, Esquilin M (2007). Racial microaggressions in everyday life: Implications for clinical practice. American Psychologist.

[CR90] Swaminathan H, Gifford JA (1985). Bayesian estimation in the two-parameter logistic model. Psychometrika.

[CR91] Tabachnick BG, Fidell LS (2007). Using multivariate statistics.

[CR92] Tepper BJ (2000). Consequences of abusive supervision. Academy of Management Journal.

[CR93] Tepper BJ (2007). Abusive supervision in work organizations: Review, synthesis, and research agenda. Journal of Management.

[CR94] Tepper BJ, Duffy MK, Shaw JD (2001). Personality moderators of the relationship between abusive supervision and subordinates’ resistance. The Journal of Applied Psychology.

[CR95] Tepper BJ, Henle CA, Lambert LS, Giacalone RA, Duffy MK (2008). Abusive supervision and subordinates deviance. Journal of Applied Psychology.

[CR96] Tepper BJ, Percy PM (1994). Structural validity of the multifactor leadership questionnaire. Educational and Psychological Measurement.

[CR97] Tepper BJ, Simon LS, Park HM (2017). Abusive supervision. Annual Review of Organizational Psychology and Organizational Behavior.

[CR98] Thoroughgood CN, Tate BWT, Sawyer KB, Jacobs R (2012). Bad to the bone: Empirically defining and measuring destructive leader behavior. Journal of Leadership & Organizational Studies.

[CR99] Trevor CO (2001). Interactions among actual ease-of-movement determinants and job satisfaction in the prediction of voluntary turnover. Academy of Management Journal.

[CR100] Walter F, Lam CK, Van Der Vegt GS, Huang X, Miao Q (2015). Abusive supervision and subordinate performance: Instrumentality considerations in the emergence and consequences of abusive supervision. Journal of Applied Psychology.

[CR101] Wang S, Noe RA, Wang ZM (2011). Motivating knowledge sharing in knowledge management systems: A quasi-field experiment. Journal of Management.

[CR102] Widiger TA, Simonsen E (2005). Alternative dimensional models of personality disorder: Finding a common ground. Journal of Personality Disorders.

[CR103] Xiaqi D, Kun T, Chongsen Y, Sufang G (2012). Abusive supervision and LMX: Leaders emotional intelligence as antecedent variable and trust as consequence variable. Chinese Management Studies.

[CR104] Zellars KL, Tepper BJ, Duffy MK (2002). Abusive supervision and subordinates organizational citizenship behavior. Journal of Applied Psychology.

